# Interleukin-15 response signature predicts RhCMV/SIV vaccine efficacy

**DOI:** 10.1371/journal.ppat.1009278

**Published:** 2021-07-06

**Authors:** Fredrik Barrenäs, Scott G. Hansen, Lynn Law, Connor Driscoll, Richard R. Green, Elise Smith, Jean Chang, Inah Golez, Taryn Urion, Xinxia Peng, Leanne Whitmore, Daniel Newhouse, Colette M. Hughes, David Morrow, Kurt T. Randall, Andrea N. Selseth, Julia C. Ford, Roxanne M. Gilbride, Bryan E. Randall, Emily Ainslie, Kelli Oswald, Rebecca Shoemaker, Randy Fast, William J. Bosche, Michael K. Axthelm, Yoshinori Fukazawa, George N. Pavlakis, Barbara K. Felber, Slim Fourati, Rafick-Pierre Sekaly, Jeffrey D. Lifson, Jan Komorowski, Ewelina Kosmider, Danica Shao, Wenjun Song, Paul T. Edlefsen, Louis J. Picker, Michael Gale

**Affiliations:** 1 Department of Cell and Molecular Biology, Uppsala University, Uppsala, Sweden; 2 Vaccine and Gene Therapy Institute and Oregon National Primate Research Center, Oregon Health & Science University, Beaverton, Oregon, United States of America; 3 Center for Innate Immunity and Immune Disease, Department of Immunology, University of Washington, Seattle, Washington, United States of America; 4 Department of Molecular Biomedical Sciences and Bioinformatics Research Center, North Carolina State University, Raleigh, North Carolina, United States of America; 5 AIDS and Cancer Virus Program, SAIC Frederick, Inc., Frederick National Laboratory, Leidos Biomedical Research, Inc., Frederick, Maryland, United States of America; 6 Human Retrovirus Section, Vaccine Branch, National Cancer Institute at Frederick, Frederick, Maryland, United States of America; 7 Human Retrovirus Pathogenesis Section, Vaccine Branch, National Cancer Institute at Frederick, Frederick, Maryland, United States of America; 8 Department of Pathology, Case Western Reserve University, Cleveland, Ohio, United States of America; 9 Statistical Center for HIV/AIDS Research and Prevention, Vaccine and Infectious Disease Division, Fred Hutchinson Cancer Research Center, Seattle, Washington, United States of America; Emory University, UNITED STATES

## Abstract

Simian immunodeficiency virus (SIV) challenge of rhesus macaques (RMs) vaccinated with strain 68–1 Rhesus Cytomegalovirus (RhCMV) vectors expressing SIV proteins (RhCMV/SIV) results in a binary outcome: stringent control and subsequent clearance of highly pathogenic SIV in ~55% of vaccinated RMs with no protection in the remaining 45%. Although previous work indicates that unconventionally restricted, SIV-specific, effector-memory (EM)-biased CD8^+^ T cell responses are necessary for efficacy, the magnitude of these responses does not predict efficacy, and the basis of protection vs. non-protection in 68–1 RhCMV/SIV vector-vaccinated RMs has not been elucidated. Here, we report that 68–1 RhCMV/SIV vector administration strikingly alters the whole blood transcriptome of vaccinated RMs, with the sustained induction of specific immune-related pathways, including immune cell, toll-like receptor (TLR), inflammasome/cell death, and interleukin-15 (IL-15) signaling, significantly correlating with subsequent vaccine efficacy. Treatment of a separate RM cohort with IL-15 confirmed the central involvement of this cytokine in the protection signature, linking the major innate and adaptive immune gene expression networks that correlate with RhCMV/SIV vaccine efficacy. This change-from-baseline IL-15 response signature was also demonstrated to significantly correlate with vaccine efficacy in an independent validation cohort of vaccinated and challenged RMs. The differential IL-15 gene set response to vaccination strongly correlated with the pre-vaccination activity of this pathway, with reduced baseline expression of IL-15 response genes significantly correlating with higher vaccine-induced induction of IL-15 signaling and subsequent vaccine protection, suggesting that a robust *de novo* vaccine-induced IL-15 signaling response is needed to program vaccine efficacy. Thus, the RhCMV/SIV vaccine imparts a coordinated and persistent induction of innate and adaptive immune pathways featuring IL-15, a known regulator of CD8^+^ T cell function, that support the ability of vaccine-elicited unconventionally restricted CD8^+^ T cells to mediate protection against SIV challenge.

## Author summary

SIV insert-expressing vaccine vectors based on strain 68–1 RhCMV elicit robust, highly effector-memory-biased, unconventionally restricted T cell responses that are associated with an unprecedented level of SIV control after challenge (replication arrest leading to clearance) in slightly over half of vaccinated monkeys. Since efficacy among monkeys vaccinated with the effective 68–1 vaccine is not predicted by standard measures of immunogenicity, we used functional genomics analysis of RhCMV/SIV vaccinated monkeys with known challenge outcomes to identify immune correlates of protection. We found that vaccine efficacy significantly correlates with a vaccine-induced response to IL-15 that includes modulation of immune cell, inflammation, TLR signaling, and cell death programming response pathways. These data suggest that RhCMV/SIV efficacy results from a coordinated and sustained vaccine-mediated induction of innate and adaptive immune pathways featuring IL-15, a known regulator of CD8^+^ effector-memory T cell function, that cooperates with vaccine-elicited CD8^+^ T cells to mediate efficacy.

## Introduction

Human immunodeficiency virus (HIV) infection continues to be a major global health problem, with approximately 38 million people worldwide currently living with HIV. Despite the decline in new infections and the remarkable success of current antiretroviral therapy (ART) at suppressing viral replication in people undergoing treatment, there were 1.7 million new HIV infections in 2019 and nearly 700,000 AIDS-related deaths [1]. Thus, the need for a vaccine to protect against HIV infection remains high, underscoring the continued essential role of RM models of SIV infection for developing and testing HIV vaccine concepts. In this regard, the highly pathogenic SIVmac251 swarm and SIVmac239 clone have been especially high bars for prophylactic vaccine efficacy with few vaccine concepts reproducibly showing sufficient efficacy against these highly pathogenic SIVs suggesting potential for clinical translation [2–4].

Among these effective vaccine concepts is the T cell response-targeted SIV vaccine that uses vaccine vectors based on RhCMV, which elicit and indefinitely maintain high frequency, circulating and tissue-based, effector memory (EM)-differentiated SIV-specific T cell responses [5–8]. RhCMV vectors were designed to provide for an immediate effector T cell intercept of immune-evasive pathogens, so as to implement anti-pathogen immune activity prior to viral implementation of effective immune evasion [2,9]. In contrast to conventional T cell-targeted prime-boost vaccines against SIV, which elicit responses that at best suppress, but never clear SIV infection, RhCMV/SIV vector-elicited immune responses have demonstrated the ability to mediate complete arrest of mucosal-acquired SIV infection prior to establishment of a long-lived SIV reservoir, ultimately resulting in progressive decline of SIV-infected cells in tissues to the point where replication-competent SIV can no longer be detected by the most sensitive assays [7,8,10]. This remarkable “control and clear” efficacy is not, however, universal, but rather occurs in ~55% of vaccinated RMs across multiple studies, with the SIV infections in the ~45% of vaccinated, non-protected RMs showing indistinguishable viral dynamics from the unvaccinated controls [6–8].

Importantly, the RhCMV/SIV vaccine vectors manifesting this efficacy are based on the 68–1 strain of RhCMV, which developed a unique genetic rearrangement during *in vitro* passage that abrogated the function of eight distinct immunomodulatory gene products encoded in two RhCMV genomic regions (Rh157.5/.4 and Rh158-161). These gene modifications had the remarkable effect of programming both the virus- and SIV insert-specific CD8^+^ T cells induced by this vector to recognize epitopes restricted by either MHC-E or MHC-II, but not classical MHC-Ia [11–13]. Differential repair of these genes reverts epitope restriction of vector-elicited CD8^+^ T cells to MHC-Ia or MHC-Ia mixed with MHC-II, whereas another mutation of 68–1 RhCMV (deletion of Rh67) results in exclusively MHC-II-restricted CD8^+^ T cells [13,14]. Although the MHC-Ia- and/or MHC-II-restricted CD8^+^ T cell responses elicited by these other RhCMV/SIV vectors manifest similar phenotypic and functional characteristics, only the original 68–1 vector shows efficacy against SIV [13,14], strongly implicating the MHC-E-restricted, SIV-specific CD8^+^ T cells as the active adaptive component of this non-antibody-inducing vaccine. However, to date, no quantitative parameter of the 68–1 RhCMV/SIV-induced T cell responses has consistently correlated with the binary outcome (protection vs. non-protection) after SIV challenge [6–8]. These observations raise the question of whether protection mediated by MHC-E-restricted CD8^+^ T cells is stochastic–i.e., based on the chance, relative distribution of infection trajectory vs. effector cell distribution in an early, critical time window after viral entry–or based on more complex characteristics of the vaccine-elicited immune response.

Here, we performed functional genomics analyses of the RM whole blood transcriptome before and longitudinally after 68–1 RhCMV/SIV vaccination, asking whether a vaccine-induced, transcriptomic response could predict RMs with comparable vaccine-induced T cell responses that were or were not subsequently protected after SIV challenge. These analyses revealed a complex transcriptomic response to vaccination that significantly correlated with subsequent outcome of SIV challenge. This efficacy-associated response was notable for a remarkable breadth of gene expression networks linked with IL-15 signaling. Our study therefore defines gene and gene network correlates of 68–1 RhCMV/SIV vaccine efficacy, and our results indicate the 55% “all or none” protection induced by this vaccine is not stochastic, but rather is dependent on specific vaccine-induced innate and adaptive immune responses.

## Results

### Characterization of 68–1 RhCMV/SIV vector-vaccinated RM cohorts with known challenge outcome

**Fig 1:**
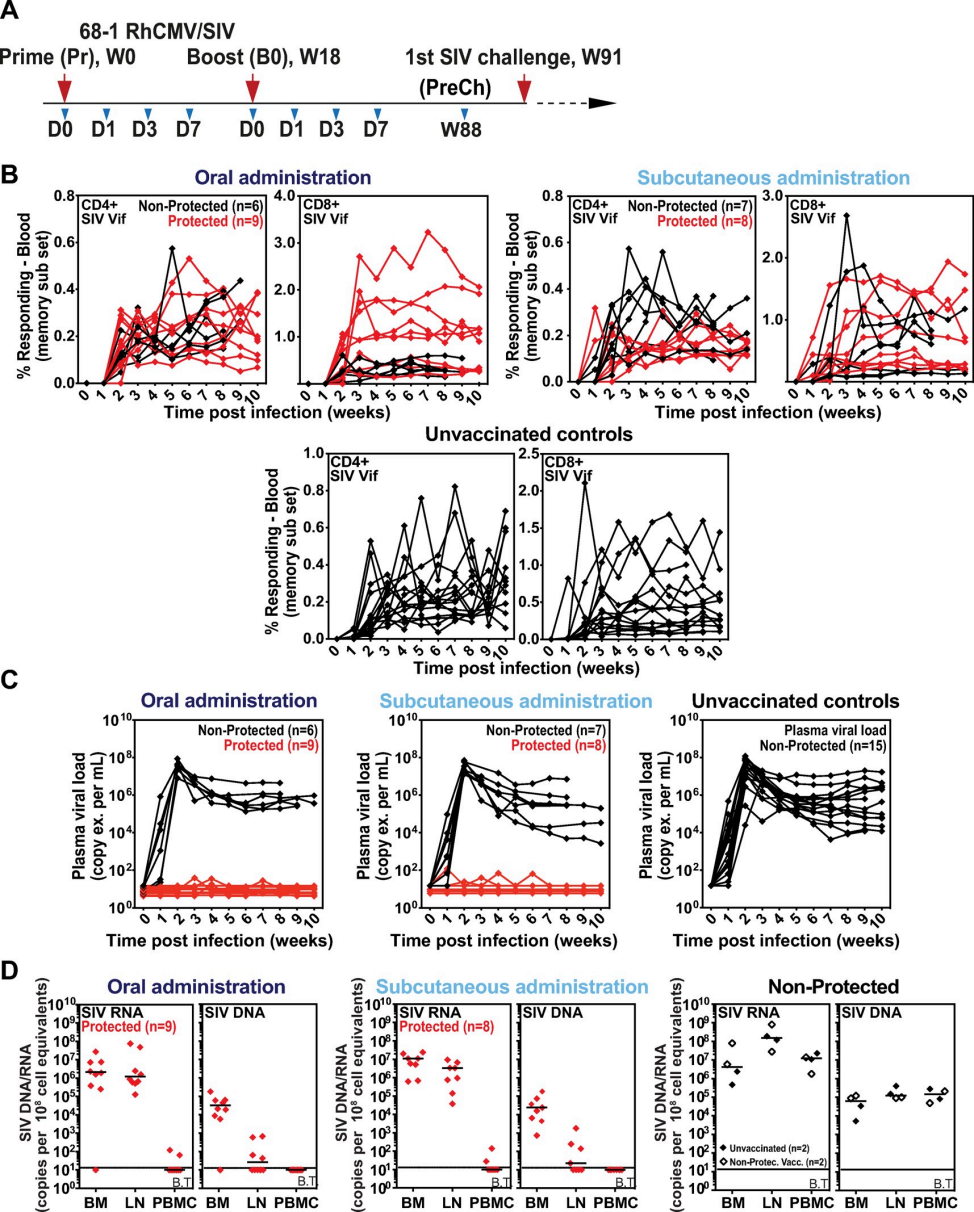
Virologic and immunologic responses in Rh/CMV vaccination and SIV challenge. **A**. Schematic of the vaccine phase of the two cohorts of RMs (n = 15 each) administered the 68–1 RhCMV/SIV vector set by either subcutaneous or oral routes at wk0 Pr and wk18 Bo, indicating time points for which whole blood samples were collected for RNAseq analysis. Repeated limiting dose SIV_mac239_ challenge was initiated at wk91. **B,C.** Assessment of the outcome of effective challenge by longitudinal analysis of the *de novo* development of SIV Vif-specific CD4^+^ and CD8^+^ T cell responses (**B**) and plasma viral load (**C**). RMs were challenged until the onset of any above-threshold SIV Vif-specific T cell response, with the SIV dose administered 2 or 3 weeks prior to this response detection considered the infecting challenge (week 0). RMs with sustained viremia were considered not protected; RMs with no or transient viremia were considered protected (6–8). **D.** Bone marrow (BM), peripheral lymph node (LN) and peripheral blood mononuclear cell (PBMC) samples from all vaccine-protected RMs and representative non-protected or unvaccinated control RMs, collected from between day 28 and day 56 post-SIV infection, were analyzed by nested, quantitative PCR/RT-PCR for cell-associated SIV DNA and RNA. The horizontal line indicates the threshold of detection (B.T. = below threshold) with data points below this line reflecting no positive reactions across all replicates. Above threshold cell-associated SIV RNA was detected in LN and BM of all protected RMs, confirming SIV infection “take”.

Two cohorts of male RMs (n = 15 each) were administered a vaccine composed of three 68–1 RhCMV/SIV vectors individually expressing SIV Gag, SIV Rev/Tat/Nef and SIV 5’-Pol, one group via a subcutaneous (subQ) route and the other via an oral route with the goal to identify fundamental correlates of vaccine protection that are independent of immunization route. Each RM was vaccinated twice, receiving a week (wk) 0 prime (Pr) and wk18 homologous boost (Bo) (**Fig 1A**). The development of SIV-specific T cells was monitored by intracellular cytokine staining (ICS), with immunogenicity in both the subQ and oral vaccine groups showing robust induction of SIV Gag-, Rev/Tat/Nef-, and Pol-specific CD4^+^ and CD8^+^ T cells (**S1A and S1B Figs)**, including CD8^+^ T cell responses to previously characterized MHC-E- and MHC-II-restricted SIV Gag supertopes–indicating, as expected, the induction of the unconventionally restricted CD8^+^ T cells; **S1C Fig**) [11,12]. These T cell responses, which were maintained through the time of challenge, manifested a striking EM bias in phenotype and function, similar to our previous analysis of T cell responses elicited by these vectors [5–8] (**S1D and S1E Figs**). Of note, there were no significant differences in these immunogenicity parameters in RMs given the vaccines via subQ vs. oral route. At wk91 after initial vaccination, these vaccinated RMs (and 15 unvaccinated controls) were subjected to repeated limiting dose, intrarectal challenge until establishment of infection “take” by detection of *de novo* T cell responses to SIVvif (an SIV antigen not included in the vaccine; **Fig 1B**), as previously described [5–8]. Analysis of plasma viral load in RMs with such infection “take” showed stringent control of viral replication in 8 of 15 and 9 of 15 RMs from the subQ and oral groups, respectively (17 total protected RMs of 30 vaccinated; 57%), with no “protected” RMs in the unvaccinated control group (**Fig 1C**). Analysis of tissue cell-associated SIV RNA and DNA load confirmed that protected RMs were indeed infected by SIV (**Fig 1D**), demonstrating that the absence of viremia in these RMs reflected immune-mediated arrest of SIV replication. Pre-specified statistical analyses of the T cell responses measured by ICS across the two vaccine cohorts found no association between magnitude or phenotype of the SIV-specific responses and protection from progressive SIV infection across the two vaccinated RM cohorts (**S2A–S2E Figs)**.

### Analysis of the whole blood transcriptome induced by subQ and oral RhCMV/SIV vaccination

**Fig 2:**
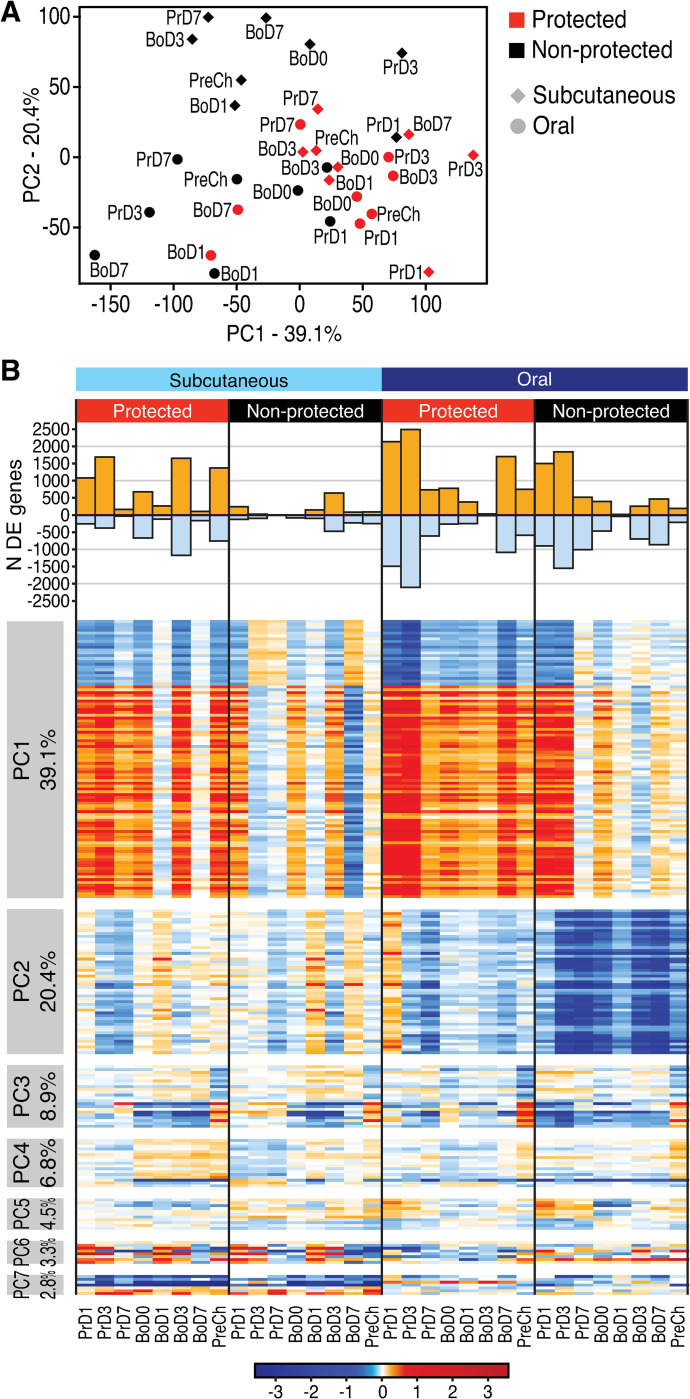
Identification of DE genes after RhCMV/SIV vaccination in protected vs. non-protected RMs. **S3 Fig. Wk0, d0 signature comparison. A**. PCA of the per-time point mean log_2_ fold-change (FC) values, using all expressed genes and averaged over the RMs within each treatment and outcome group, showing the mean log_2_(FC) of all expressed genes per time point in oral (circles) and subcutaneous (diamonds) for protected (red) and non-protected (black) RMs. **B.** Upper panel: number of DE genes per time point in each group. Lower panel: Heatmap showing genes most associated with each PC, for the first 7 principal components. Percent variance explained by each PC is shown at left. Prime, boost, and pre-challenge time points are shown at bottom.

To determine whether other parameters of the vaccine response might correlate with protective immunity across the vaccinated RM groups, we performed longitudinal global transcriptomic profiling of mRNA expression in whole blood samples collected prior to prime (Pr) and boost (Bo) RhCMV/SIV vaccinations (d0 of wk0 and wk18, respectively), at day (d)1, d3 and d7 following Pr and Bo, and at wk 88, 3 wks prior to initiating SIV challenge (see **Fig 1A**). We first evaluated the gene expression responses to vaccination (change from pre-vaccination baseline) by performing a principal component analysis (PCA) on the per-timepoint mean log2 fold-change (FC) values, using all 12,183 expressed genes and averaged over the RMs within each treatment and outcome group by time (**Fig 2C**). While this analysis did not explicitly account for the outcome, the top two components, PC1 and PC2, turned out to segregate the animals into protected and non-protected groups. To further evaluate these differences, we defined the set of genes with statistically significant differential expression (DE) at any post-vaccination time point compared to pre-vaccination baseline in any of the four RM subgroups defined by administration route and protection outcome, using bioinformatics analyses and linear modeling (**Fig 2B and S1 Table**). These analyses suggested that DE genes associated with variation in PC1 are relevant for establishing a vaccine protective signature. PC1 DE gene expression changes occurred rapidly after vaccination (d1 in both cohorts), with highest numbers and absolute levels of gene expression change from baseline found in the orally immunized cohort 3d after the priming immunization. The expression of these DE genes was variably reduced thereafter, but was relatively maintained in the protected RMs, with the expression level change increasing to a greater degree in the protected group following vaccine boost. Importantly, absolute gene expression changes of the PC1 DE genes persisted through the pre-challenge (PreCh) time point in the protected RMs, whereas non-protected RMs failed to maintain or re-establish this signature by the PreCh time point.

### Gene network correlates of protection

**Fig 3:**
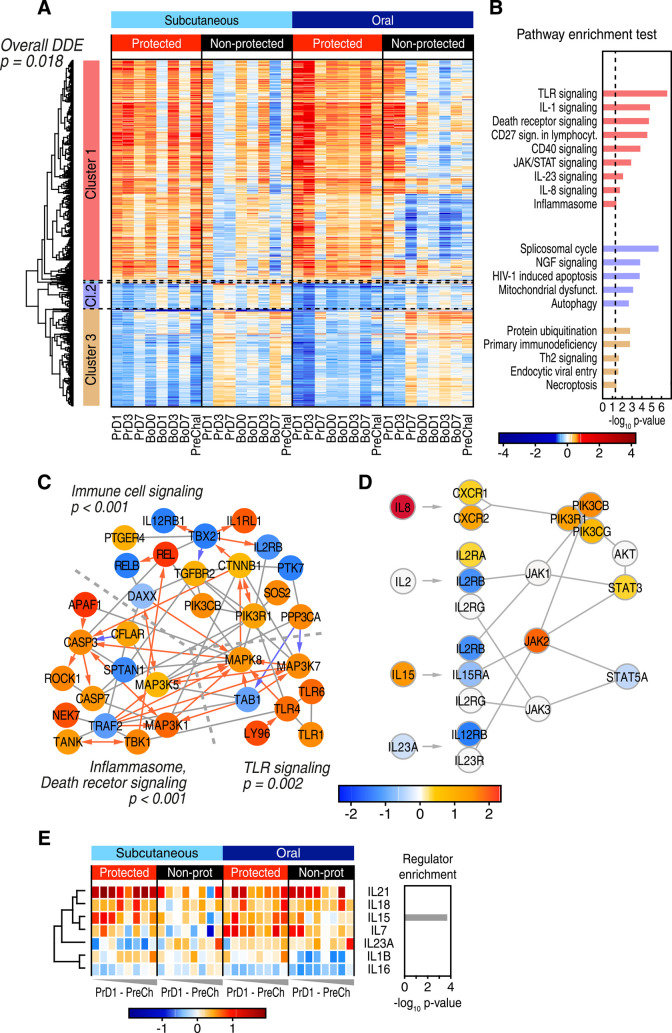
Gene and pathway correlates of protection. **A**. Heatmap showing all DDE genes. Three clusters were defined using hierarchical clustering. **B**. Ingenuity pathway analysis of the three major DDE gene clusters. **C**. Network of direct physical interactions of encoded proteins (indicated by grey connector line) between major enriched immune pathways with red and blue arrows indicating activating and inhibitory interactions, respectively. P-values for the association of each pathway with vaccine protection are shown and are based on permutation testing. **D**. Network overview of JAK-STAT signaling in enriched interleukin pathways. **E**. Heatmap of gene expression changes for expressed interleukin genes (**left**), and their enrichment as upstream regulators of DDE genes using Ingenuity analysis (**right**).

To identify gene expression correlates of protection and their functional regulatory networks, we determined the genes showing significant differential change-from-baseline expression between protected and non-protected groups across the time course, designated as DDE genes. The 1,763 DDE genes identified (**S2 Table**) capture the vaccine response signature that varied between the protected and non-protected outcome groups (**Fig 3A)**. Using permutation testing we verified that sum of the absolute magnitude of differential expression between protection outcomes was significant (p = 0.018; see Methods), thus linking DDE gene expression with vaccine protection. To identify specific response pathways and networks among the DDE genes that define vaccine protection, we first performed clustering analysis and found three major clusters containing either mostly induced/up-regulated genes (Cluster 1) or suppressed/down-regulated genes (Clusters 2 and 3) in the protected RMs across both vaccine groups (**Fig 3A**). The vaccine-induced gene cluster was associated mainly with innate immune pathways spanning TLR signaling and cytokine response pathways, inflammasome/cell death signaling, and included a set of immune cell signaling genes (**Fig 3B**).

Pathway analyses of DDE gene network linkage identified death receptor signaling/inflammasome network interactions with a TLR network and immune cell signaling network, each significantly linked with vaccine protection (**Fig 3C**). These networks connect through a mitogen-activated protein (MAP) kinase signaling axis marked by *PPP3CA*/calcineurin and known interacting partners including *MAP3K7*/TAK1, *MAP3K5*/ASK1 [15–17], and *MAP3K1*/MEKK1. We defined the immune cell signaling network based on the DDE enrichment of mRNA encoding *IL-2RB*/CD122, a receptor subunit of both IL-2 and IL-15 [18]. This network includes *TBX21/*T-bet within an axis of innate immune and inflammatory signaling proteins RELA-B/NF-kB [19], TBK1 [20], and TANK [21] linked with TLR signaling [22] (**Fig 3C**). Included in this network are phosphatidylinositol responsive kinase (PI3K) subunits (*PIK3R1* and *PIK3CB*) [23] and *CTNNB1*/beta-catenin linked with *PTK7*/protein tyrosine kinase 7 [24,25]. Notably, these factors mediate intracellular signaling, cell-cell communication, and interaction with the Wnt signaling pathway implicated in T cell regulation [26–29]. Together, these gene networks and their differential regulation of gene expression collectively define a vaccine protection gene network signature in the blood of protected RMs.

We also found that JAK-STAT cytokine signaling was enriched within the DDE gene signature in the absence of typical type I interferon stimulated genes (ISGs), suggesting involvement of different interleukin signaling programs (**Fig 3D**). We therefore interrogated our gene expression data sets for expression levels of all interleukins, identifying only IL-1β, IL-7, IL-15, IL-16, IL-18, IL-21, and IL-23A as being expressed at one or more time points within the vaccine cohorts. Among these, IL-15 differential expression was both enriched in protected RMs (p = 0.032) and identified as a significant upstream regulator of the vaccine protection-associated DDE signature (**Fig 3E**). The increased upregulation of IL-15 expression in protected, compared to nonprotected, RMs was accompanied by downregulation of IL-15 receptor subunits and their JAK-STAT signaling components relative to the other interleukins. Downregulation of cytokine receptor expression is a specific marker of active cytokine signaling [30], suggesting that an active IL-15 cytokine signaling pathway is a component of the DDE protection signature (see **Fig 3D**).

### Protection signature links with IL-15 signaling

**Fig 4:**
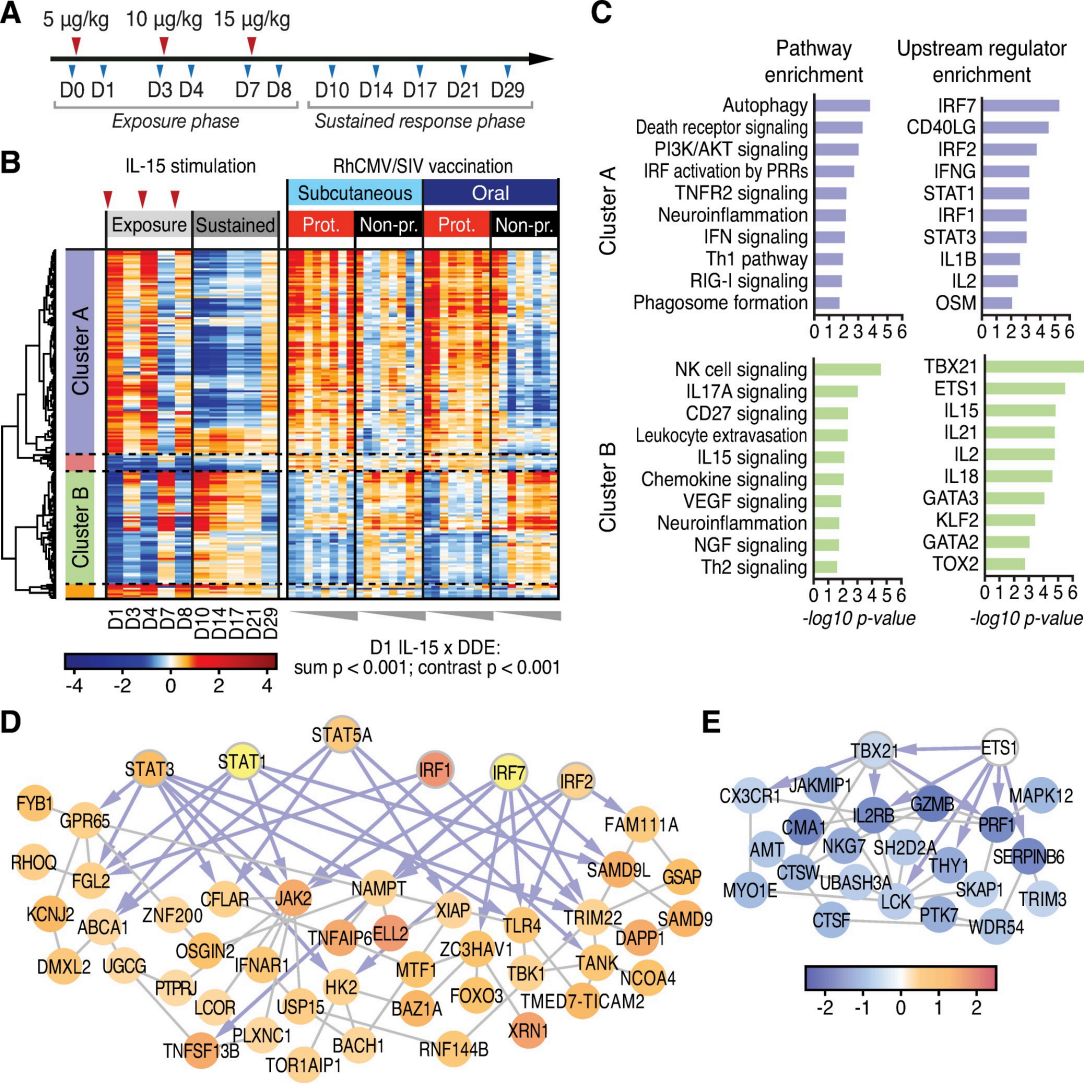
The IL-15 response links with correlates of vaccine protection. **A**. Study design–RM treatment with rRh-Het-IL-15. Red arrowheads indicate IL-15 treatment. **B**. Heatmap of DDE gene correlates of protection regulated by IL-15 (IL-15 DE genes on d1 post-administration). P-values, shown below the heatmap, are highly significant for both the sum statistic and the contrast statistic differing across protection outcomes. The sum statistic is the average over time of the sum of the absolute change-from-baseline expression, and the contrast statistic is the average over time of the sum of the change-from-baseline expression of IL-15 d1 up-regulated genes minus the sum over the IL-15 d1 down-regulated response genes (see Methods). **C**. Ingenuity pathway and upstream regulator enrichment analyses for gene cluster A and B from panel B. **D,E.** *De novo* network of IL-15 response genes from Cluster A (**D**), and Cluster B (**E**) were constructed in GeneMania. Transcription factor nodes are indicated by thick borders with connections shown as gray lines and blue arrows, showing co-expression interactions (GeneMania) and direct transcription factor-target interactions (Ingenuity), respectively.

IL-15 plays a major role in cellular immune programming, supporting memory T cell and NK cell activation, homing, homeostasis, both effector differentiation and function, and in particular controlling the activity of circulating and tissue resident CD8^+^ EM T cells [31–35]. IL-15 activates signaling through the β chain and common γ chain heterodimer of the IL-2 receptor, either as a soluble heterodimer with the α chain of the IL-15 receptor (IL-15Rα) or through trans-presentation by cells expressing IL-15Rα [36–38]. Mimicking this process, recombinant, purified heterodimeric IL-15/IL-15Rα (rRh-Het-IL-15) is a potent immune therapeutic to induce IL-15 signaling [39–41]. To further define the whole blood gene expression signature directed by IL-15 *in vivo* and to evaluate the breadth of the IL-15 response in 68–1 RhCMV/SIV vaccinated RMs, we conducted transcriptomic analysis on whole blood samples from a separate cohort of five unvaccinated RMs treated with rRh-Het-IL-15 in a dose-escalating fashion (5, 10, and 15 μg/kg at d0, d3 and d7 respectively) with blood collection through d29 (**Fig 4A**). We identified DE genes responding to rRh-Het-IL-15 treatment *in vivo* across this 29d time course (**S3 Table**) in which heterodimeric IL-15 administration rapidly altered gene expression within one day, followed by a homeostatic reset of expression levels two days later (**Fig 4B, left panels**). We used the d1 post IL-15 administration DE genes to interrogate the DDE signature of the vaccinated RMs for evidence of an embedded IL-15 response. The d1 DE gene set was selected to reflect the direct response of the RMs to IL-15 prior to the onset of homeostatic regulation. We identified multiple co-expression clusters within the intersecting gene set of 186 genes (**Fig 4B, S4 Table**). Pathway analyses of each cluster revealed an overlap of the response to rRh-Het-IL-15 with several of the immunological pathways identified in the DDE analysis, including up-regulated pathways (cluster A) of PI3K signaling, death receptor signaling, innate immune signaling, and immune cell signaling modules (**Fig 4C**). These networks link to interferon regulatory factor (IRF) and STAT transcription factors as major upstream regulators responding to IL-15 signaling. Moreover, we identified acutely down-regulated IL-15 response genes that were also components of the DDE protection signature (see **Fig 4C**, cluster B). Notably, among genes showing acute downregulation by rRh-Het-IL-15 treatment were pathways regulated by *TBX21*/Tbet, consistent with the immune cell and cytokine signaling DDE signatures (see **Fig 3C and 3D**).

To identify interaction between IL-15 regulated genes within the DDE protective signature, we built *de novo* gene interaction networks using GeneMania [42], basing network construction on genes within each cluster of our intersecting dataset. Among genes up-regulated by rRh-Het-IL-15 treatment within the DDE vaccine protection signature, we identified multiple pathways involved in immune activation including TLR signaling, innate immune activation, and death receptor signaling, each linked to specific transcription factor nodes (IRF1, IRF2, IRF7, STAT1, STAT3, and STAT5). As these IRFs and STATs are also prominently linked to innate immune signaling [43], these results together reveal a remarkable breadth of signaling crosstalk in immune programming wherein IL-15 signaling intersects both innate and adaptive immune pathways in building the DDE protection signature, likely reflecting a cascade of direct and indirect IL-15 signaling actions (**Fig 4D and 4E**). Of note, we identified linkage of IL-15 down-regulated genes with the DDE immune signaling network (see **Figs 3C and 4E**) including *TBX21*, *IL2RB*, and *PTK7*. Most importantly, the IL-15xDDE gene set (acute IL-15 response DE genes at d1, filtered to DDE genes) was significantly linked with vaccine protection (p < 0.001), using the sum statistic described above. We then evaluated these IL-15xDDE genes for consistency with the directionality of their IL-15 d1 DE response using a contrast statistic (sum of up genes—sum of down genes, see Methods), and found that this statistic was also highly significant (p < 0.001). These analyses identify IL-15 as a major regulator of the DDE signature underlying RhCMV/SIV vaccination outcome and demonstrate linkage of IL-15 responsive genes with vaccine efficacy.

### Conserved IL-15 response significantly links with vaccine protection in validation cohort analysis

**Fig 5:**
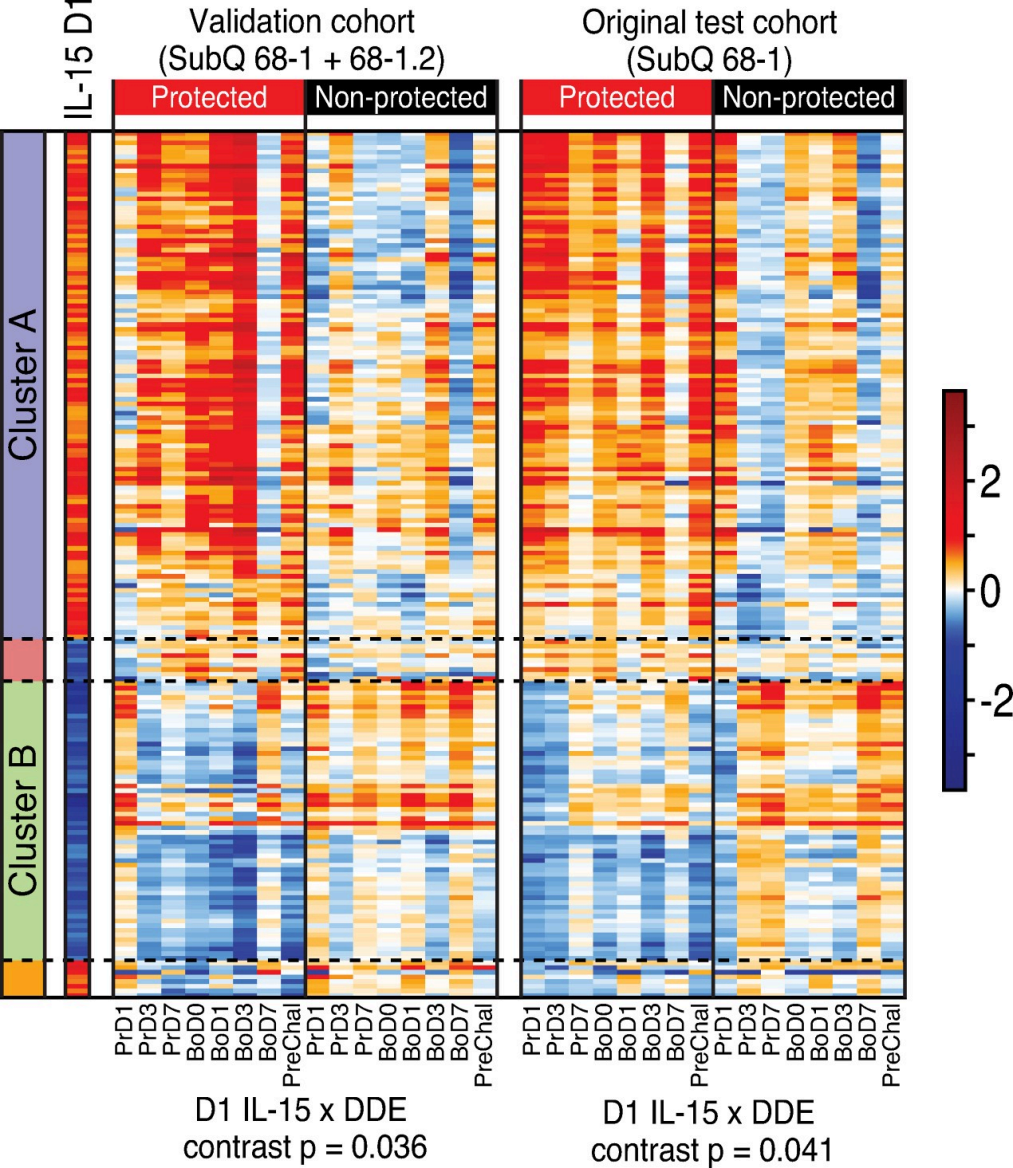
Validation of the IL-15 response signature of protection. Heatmaps comparing the IL-15 response signature (overlap of DDE and day 1 IL-15 DE genes as shown in **Fig 4B**) in the validation cohort (left panel; 68–1 + 68–1.2 RhCMV/SIV vaccinated; 6 of 15 protected) vs. the original subQ 68–1 RhCMV/SIV vaccinated cohort (right panel; 8 of 15 protected). P-values, shown below each heatmap, are significant for both cohorts for the permutation test evaluating the contrast statistic across protection groups, which had been pre-specified for validation. The contrast statistic is the average over time of the sum of the change-from-baseline expression of IL-15 d1 up-regulated genes minus the sum over the IL-15 d1 down-regulated response genes (see Methods).

We next evaluated the whole blood gene expression signature in an independent validation cohort of 15 RMs that had been subQ vaccinated with a combination vaccine including the same 68–1 RhCMV/SIV vaccine set used in the RMs described above and a variant 68–1.2 RhCMV/SIV vaccine set with the same SIV inserts (**S5 Table)**. The 68–1.2 RhCMV/SIV vector is repaired for pentameric complex expression and therefore is programmed to elicit MHC-Ia-restricted CD8^+^ T cell responses, resulting in these vaccinated RM having both conventionally (MHC-Ia) and unconventionally (MHC-E and MHC-II) restricted SIV-specific CD8^+^ T cell responses [13]. 68–1.2 RhCMV/SIV vectors are not protective, but they do not abrogate 68–1 RhCMV/SIV vector-mediated protection, and 6 of the 15 vaccinated RMs in this validation cohort manifested stringent viral control after SIV challenge [13]. Although the 68–1+68–1.2 combination vaccine is quite different than the 68-1-only vaccine, the protection phenotype in these RMs (e.g., viral replication-arrest) was identical to our 68-1-only vaccinated RM cohorts, suggesting that protection-critical signaling of gene expression such as the DDE and IL-15 signatures (see **Fig 4**) might be preserved in protected animals receiving this combination vaccine. The validation 68–1+68–1.2 cohort was studied in parallel with the subQ and oral 68–1 vaccinated cohorts, and sample collection, processing and RNAseq analysis were performed identically. Comparison of the post-vaccination change-from-baseline expression pattern of the IL-15-regulated genes shown in **Fig 4B** (intersection of DDE and day 1 rRh-Het-IL-15 DE) in protected vs. non-protected RMs in the validation cohort and the subQ 68–1 vaccinated cohort (also n = 15) revealed a very similar pattern of gene expression within the blood of protected RMs in both cohorts showing a more pronounced and durable IL-15 response to vaccination than the non-protected RMs (**Fig 5; S4 and S6 Tables**). In keeping with this outcome, our pre-specified analysis evaluating the vaccine response in protected animals in both cohorts showed a greater contrast across the directional (up and down-regulated) IL-15 responses (filtered to DDE genes) than in non-protected animals and was significantly associated with vaccine protection (p = 0.036), similar to the result when evaluating this contrast in the equally sized subQ group alone (p = 0.041). These results validate the IL-15/DDE signature as a significant gene expression program linked with RhCMV/SIV vaccine efficacy.

### DDE and IL-15 pathway gene signatures from baseline link with vaccine protection

**Fig 6:**
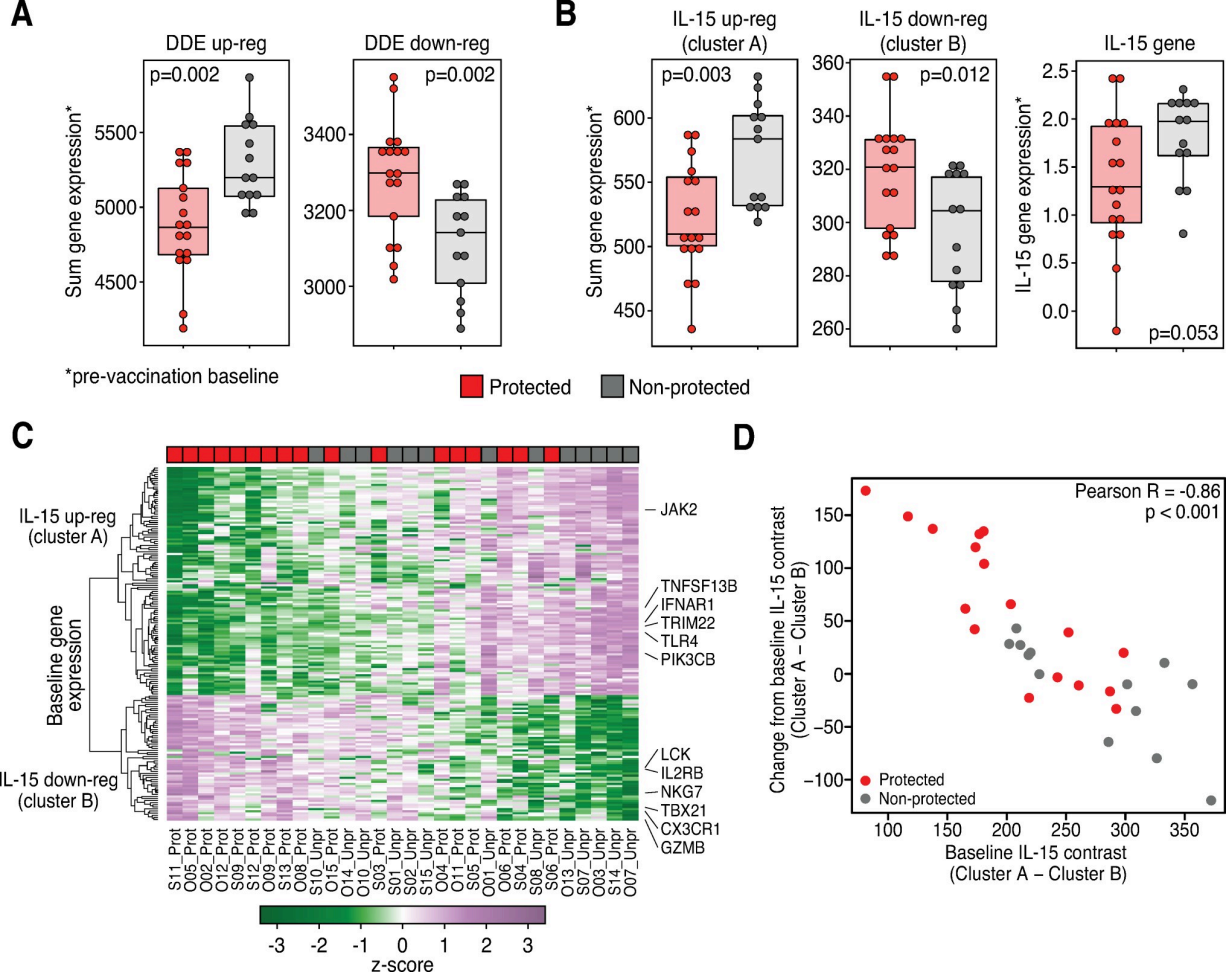
Baseline evaluation of DDE and IL-15 response signature of protection. Box plots and heat map comparing normalized expression values at baseline across protection outcomes. **A**. Box plots show sum of normalized expression counts over the genes identified as DDE up-regulated or down-regulated across protection outcome groups (**S2 Table**) with unadjusted Wilcoxon p-values shown. **B**. Analogous results for the IL-15 d1 clusters A (up) and B (down) at baseline (left two panels) and IL-15 gene expression (right panel). **C**. Heat map showing the IL-15 response genes in the subQ and oral cohort RMs at baseline. The positions of selected genes are indicated at right. **D**. Scatter plot showing the IL-15 cluster A minus cluster B contrast for each individual animal, at baseline vs. the change-from-baseline average over time post-vaccination (Pearson correlation R and p-value shown); protected RMs are shown in red; non-protected in gray.

To determine if the gene expression signature observed in response to vaccination is also predictable from baseline (pre-vaccination), we applied the analogous primary statistical test that we used to identify change-from-baseline DDE genes to the baseline transcriptome of each RM in the original subQ and oral vaccine cohorts. We found that across all expressed genes, there was significant differential expression at baseline across protection groups (p = 0.034). We also found that the baseline expression of the DDE up genes (defined as those genes with significantly *greater* change-from-baseline among protected vs. unprotected RMs; **S2 Table**) is significantly *lower* among protected relative to unprotected animals, and that the baseline expression of the DDE down genes is significantly *higher* (**Fig 6A**). A similar pattern is seen among the d1 IL-15xDDE cluster A and B gene sets and their directionality (see **Fig 4B**), with expression of the up-regulated cluster A genes *lower* and the down-regulated cluster B genes *higher* in magnitude (**Fig 6B**, left two panels) at baseline in the vaccine-protected animals relative to the unprotected animals. In keeping with these findings, the normalized baseline expression of the IL-15 gene itself was lower in protected RMs and nearly significant in differentiating outcomes at baseline by a simple Wilcoxon test (p = 0.053; **Fig 6B**, right panel). A Z-score heat map of the IL-15 response genes within the DDE vaccination signature at baseline illustrates the relationship between the magnitude of this baseline IL-15 response gene expression and vaccine protection (**Fig 6C and S7 Table**). Importantly, there was a very tight correlation (p < 0.001) between the pre-vaccination baseline expression of the IL-15xDDE cluster A and B gene sets and their post-vaccination change-from-baseline expression of the same genes (**Fig 6D**), strongly suggesting that the ability of the vaccine to induce and maintain IL-15 signaling depends on the level of activity of this signaling prior to vaccination, with more robust post-vaccination signaling and efficacy associated with IL-15 (and overall DDE) signaling pathway quiescence at baseline. We found that the overall predictive value of the IL-15 signaling change-from-baseline contrast statistic after vaccination is 77% (AUC 0.78) for the oral and subQ cohorts (**Figs 6D and S3**), but as shown in these figures, the association of IL-15 signaling status with outcome at either end of the signaling spectrum is nearly perfect, with outcome uncertainty a feature of an intermediate IL-15 signaling response. Thus, while the association of IL-15 signaling and outcome is quite strong, it is likely that other yet-to-be elucidated factors, including those within the identified immune cell signaling, TLR, and cell death/inflammasome modules that might operate independently of IL-15, contribute to 68–1 RhCMV/SIV vaccine efficacy at intermediate levels of IL-15 stimulation.

## Discussion

The pattern of efficacy resulting from 68–1 RhCMV/SIV vector vaccination in RMs is unprecedented, with an early complete replication arrest of nascent SIV infection occurring in slightly over half of vaccinees (protection), whereas the remaining vaccinees manifest primary infection SIV dynamics similar to unvaccinated controls (non-protection). Previous work associated the early effective intercept of nascent SIV with the characteristic effector-memory-biased T cell response elicited by RhCMV [5–8] and more recent work has strongly linked efficacy with the also unprecedented MHC-E-restricted CD8^+^ T cell response that results from the unique strain 68–1 RhCMV genotype [12–14]. However, the very consistent, binary 50–60% efficacy across multiple studies has not been explained, as no standard immunologic parameter has been consistently correlated with protection after challenge, including both this study and prior studies [6–8]. Indeed, given the fact that 68–1 RhCMV/SIV vector-mediated protection is determined very early by pre-existing immune responses without anamnestic expansion, it was possible that protection was simply stochastic, based on available immune responses at the random local sites involved in initial infection. Here, using transcriptomic analysis, we provide evidence that in contrast to this “random landing” hypothesis, 68–1 RhCMV/SIV vector-mediated efficacy is not primarily stochastic, but rather it appears to depend on the ability of the vaccine to generate specific innate and adaptive immune responses. We identified a whole blood gene expression signature that strongly correlates with, and very likely underlies RhCMV/SIV vaccine efficacy in RMs. This signature encompasses functional pathways/modules consisting of immune cell signaling (defined in this study), TLR signaling, and inflammasome/cell death signaling, and in particular IL-15 signaling that all significantly correlate with vaccine efficacy. The magnitude and persistence of gene expression changes following vaccination are significantly higher throughout the vaccine phase in RMs destined for protection after challenge, with the IL-15 response signature being a correlate of a vaccine efficacy across 3 RM cohorts that differed in route of vaccination (oral vs. subQ) and the nature of the vaccine itself (68–1 vector set vs. 68–1+68–1.2 vector set).

Bioinformatic analyses identified IL-15 as an enriched upstream regulator of the DDE signature linked with vaccine protection, and this was confirmed by direct analysis of the *in vivo* response to IL-15 administration in RMs. Although many immune-related signaling pathways are so broadly functional that their specific contributions to a given immune response can be difficult to discern, this is not the case for IL-15. IL-15 is produced by myeloid cells, including dendritic cells and monocyte/macrophages, and primarily acts to maintain population homeostasis and to regulate the effector function, activation thresholds and homing behavior of memory (particularly effector memory) T cells and NK cells [31–35]. Since adaptive (SIV-specific) cellular immunity is required for RhCMV/SIV-based vaccine efficacy [7], the most likely target of this cytokine in our protected RMs are the unconventionally MHC-E-restricted, effector-memory, SIV-specific CD8^+^ T cell responses that, as indicated above, are both uniquely elicited by this vaccine and strongly associated with its efficacy [13, 14]. We would thus speculate that IL-15 signaling might contribute to efficacy by modulating the differentiation of these critical SIV-specific CD8^+^ T cells, enhancing their effector function and/or modulating their epitope sensitivity, and/or driving their migration to tissue sites that support early SIV replication. In keeping with this, our functional genomics analyses of the whole blood response shows that IL-15 induces gene networks linked to lymphocyte activation, migration and homing. However, IL-15 also regulates the homeostasis, function and behavior of other cell types, notably CD4^+^ memory-effector T cells and NK cells, and we cannot currently rule out a contribution of these cell types to determining protection vs. non-protection, either by modulating their effector function/homing behavior, as suggested for CD8^+^ T cells, or for CD4^+^ memory cells, by modulating their ability to support SIV infection.

We also found that IL-15 signaling manifested a broad interface with innate immunity linked with IRF transcription factors and STAT signaling, and in particular, driving or linking with TLR and inflammasome/cell death gene expression networks that also correlated with efficacy. It is notable that each implicated network links to the others through mRNAs encoding upstream MAP kinases (MAPK8/c-jun N-terminal kinase, MAP3K7/TAK1, MAP3K5/ASK1, and MAP3K1/MEKK1), thus revealing a MAP kinase signaling axis responsive to the 68–1 RhCMV/SIV vaccine. MAP3K7/Tak1 is a facilitator of calcineurin-NFAT signaling that directs NFATc1 nuclear translocation, NFAT transcriptional activation and cell proliferation [15] wherein these actions may serve to program immune effector cells (e.g., MHC-E-restricted CD8^+^ T cells) to support vaccine protection. MAPK8/c-jun N-terminal kinase and MAP3K1/MEKK1 are also implicated in WNT signaling that supports dendritic cell maturation [44]. WNT signaling is mediated in part by CTNNB1/beta-catenin, which is also a DDE gene, and can serve to program CD8^+^ T cell development and effector functions in processes governed by PIK3 signaling in T cells [45–47]. These analyses raise the possibility that CTNNB1/beta-catenin regulation MAP kinase and PI3K cascades serve crucial immune programming functions for 68–1 RhCMV/SIV vaccine-induced immunity, and therefore that the broad signaling cross talk plays a direct role in 68–1 RhCMV/SIV efficacy. Developing approaches to facilitate this network crosstalk and gene regulation could offer strategies to enhance vaccine efficacy.

Our analysis of serial, dose-escalating IL-15 administration *in vivo* shows that IL-15 directs a rapid alteration of the blood transcriptome within 1 day to both induce and suppress specific gene expression from pretreatment baseline levels with similarly rapid homoeostatic "resetting" of the blood gene expression profile 2 days later. Repeated IL-15 dosing and dose escalation generated a decreased response by day 8 post-initial treatment even in the continued presence of high IL-15 levels, followed by homeostatic regulation of gene expression through the remaining 21 days of the time course. This rapid resetting of the IL-15 inductive response after the initial 2 doses, and the refractory period after the 3^rd^ highest dose suggests the development of a counter-regulated “cytokine tolerant" phenotype. Since the IL-15 signaling signature that tracks with vaccine efficacy is persistent, it is likely that the level of IL-15 that drives this efficacy-associated signature is low, achieving a balance between inductive and counter-regulatory pathways that provides for long-term maintenance of IL-15 signaling above the pre-vaccination baseline. In keeping with this importance of establishing a precise balance between IL-15 inductive and counter-regulatory pathways, we found that prior to vaccination, RMs destined for protection after vaccination manifested a significantly lower level of directional expression of IL-15 response genes than RMs destined for non-protection after vaccination, suggesting that quiescence of the IL-15 signaling pathway at baseline contributed to establishment of higher, persistent levels of IL-15 signaling after vaccination. We hypothesize that higher pre-vaccination IL-15 activity in RMs destined for non-protection might impart cytokine tolerance, restricting the IL-15 signaling response to vaccination in terms of both magnitude and persistence. This phenotype of cytokine tolerance is analogous to what can occur in patients undergoing interferon-alpha therapy for chronic hepatitis C Virus infection, in which high pre-treatment levels of hepatic interferon resulted in innate immune tolerance and lack of hepatic ISG induction that rendered an overall nonresponse to treatment [48]. The basis for higher baseline IL-15 signaling in RMs destined for non-protection will be important to determine for development of strategies to counter this tolerance phenotype and enhance efficacy of 68–1 RhCMV/SIV vaccines. If the elevated baseline IL-15 signaling is simply a response to an ongoing transient, unrelated immune response, it might be possible enhance vaccine efficacy by withholding vaccination until quiescence of such responses. On the other hand, if the elevated baseline IL-15 signaling reflects an intrinsically, perhaps genetically determined, higher IL-15 activity set point, effective vaccination might require pre-treatment with signaling inhibitors or other strategies that would artificially provide the IL-15 pathway quiescence needed for effective vaccination. Strategies to deliver a low, sustained production and response to IL-15 after vaccination might also serve as effective adjuvant approaches to enhance vaccine efficacy. Of note, we posit that it is highly unlikely that differences among RMs in vector spread and persistence account for the post-vaccination immune signaling heterogeneity, as typical 68–1 RhCMV/SIV protection is observed with pp71-deleted 68–1 RhCMV/SIV vectors that manifest ~1000-fold reduction in *in vivo* spread compared to the vectors used in this study [8].

In conclusion, these studies provide important new insight into the unique “all or none” viral replication arrest efficacy of 68–1 RhCMV/SIV vectored vaccines, indicating that not only is this efficacy associated with elicitation of SIV-specific MHC-E-restricted CD8^+^ T cells, as previously described [13,14], but in the setting of such responses, it is also strongly correlated with a specific, vaccine-induced innate and adaptive immune signaling signature. Understanding the biologic interaction of these two strong efficacy correlates will likely allow for refining and improving the predictive signature, but more importantly, will almost certainly reveal the fundamental biologic mechanisms underlying SIV replication arrest, which could have far-reaching implications for both vaccine and non-vaccine approaches to control of SIV/HIV infection. In the shorter term, the discovery that the development the post-vaccination protective innate/adaptive immune signaling signature depends on the pre-vaccination set point of the same pathways offers the possibility of enhancing efficacy using therapeutic interventions that modulate these set points and the initial innate response to vaccination. Finally, it is highly likely that recapitulation of 68–1 RhCMV/SIV efficacy in people will require an orthologous HCMV/HIV vaccine to recapitulate of the same immune mechanisms in people, and thus, both the MHC-E-restricted CD8^+^ T cell and innate/adaptive immune correlates will be crucial for guiding ongoing clinical development of such a vaccine, providing immunologic surrogates of potential efficacy in early, vaccine safety-focused Phase I and IIa clinical trials [49,50].

## Methods

### Ethical statement

RM care and all experimental protocols and procedures were approved by the ONPRC Institutional Animal Care and Use Committee. The ONPRC is a Category I facility. The Laboratory Animal Care and Use Program at the ONPRC is fully accredited by the American Association for Accreditation of Laboratory Animal Care and has an approved Assurance (#A3304-01) for the care and use of animals on file with the NIH Office for Protection from Research Risks. The ONPRC adheres to national guidelines established in the Animal Welfare Act (7 U.S.C. Sections 2131–2159) and the Guide for the Care and Use of Laboratory Animals (8th Edition) as mandated by the U.S. Public Health Service Policy.

### Rhesus macaques

The experiments reported in this study used a total of 65 purpose-bred male and female RMs (*M*. *mulatta*) of Indian genetic background, including 15 RMs assigned to each of four vaccine groups (oral 68–1 vaccination, subQ 68–1 vaccination, an unvaccinated control group and a subQ 68–1 + 68–1.2 vaccinated test group), and 5 RMs administered rRh-Het-IL-15 for the IL-15 blood signature assessment. The unvaccinated and subQ vaccinated RM cohorts are also reported in Malouli, *et al* [13]. At assignment, all study RMs were free of cercopithecine herpesvirus 1, D-type simian retrovirus, simian T-lymphotrophic virus type 1, and *Mycobacterium tuberculosis*, but were naturally RhCMV-infected. All study RMs were housed at the Oregon National Primate Research Center (ONPRC) in Animal Biosafety level 2 (vaccine phase) and level 2+ (challenge phase) rooms with autonomously controlled temperature, humidity, and lighting. Study RMs were both single- and pair-cage housed. Animals were only paired with one another during the vaccine phase if they belonged to the same vaccination group. All RMs were single cage-housed during the challenge phase due to the infectious nature of the study. Regardless of their pairing, all animals had visual, auditory and olfactory contact with other animals. Single cage-housed RMs received an enhanced enrichment plan that was designed and overseen by RM behavior specialists. RMs were fed commercially prepared primate chow twice daily and received supplemental fresh fruit or vegetables daily. Fresh, potable water was provided via automatic water systems. Physical exams including body weight and complete blood counts were performed at all protocol time points. RMs were sedated with ketamine HCl or Telazol for procedures, including oral and subQ vaccine administration, venipuncture, and SIV challenge.

All vaccinated RMs in this study were administered a single set or 2 sets of three RhCMV/SIV vectors (68–1 backbone or 68–1 + 68–1.2 backbones), individually expressing SIV Gag, Retanef (Rev/Tat/Nef) and 5’-Pol (see below), either orally or subcutaneously at a dose of 5x10^6^ plaque-forming units per vector, with a second identical vaccination given 18 wks after primary vaccination. At the end of vaccine phase, all vaccinated and unvaccinated RMs were SIV challenged by repeated (every 2–3 wks) intra-rectal administration of limiting dose (100–300 focus-forming units) SIV_mac239X_ (described below) until take of infection (onset of sustained plasma viremia and/or *de novo* development of CD4^+^ and CD8^+^ T cell responses to SIV Vif), at which time challenge was discontinued, as previously described [6–8]. For *in vivo* determination of the transcriptomic response to IL-15, a cohort of five RMs were treated with rRh-Het-IL-15 prepared by Drs. George Pavalakis (National Cancer Institute, USA) and Jeff Lifson (Frederick National Laboratory, USA) in a dose-escalation manner as follows: 5, 10, and 15 μg/kg at D0, D3 and D7 respectively. Whole blood was collected in PAXgene tubes at d 1, 3, 4, 7, 8, 10, 14, 17, 21, and 29 for transcriptomic analysis. RNA samples from *in vivo* rRh-Het-IL-15 treatment were processed for RNAseq transcriptomic analyses as described below.

### Vectors and viruses

Construction and characterization of the 68–1 and 68–1.2 RhCMV/SIV vectors, including RhCMV/SIV_Gag_, RhCMV/SIV_Retanef(Rev/Tat/Nef)_ and RhCMV/SIV_5’-Pol_ have been previously described [6,7,12,13]. Vector stocks were generated on telomerase-immortalized rhesus fibroblasts. SIV transgene expression was confirmed by immunoblot and all virus stocks were analyzed by next generation sequencing before *in vivo* use. Virus titers were determined by 50% tissue culture infective dose endpoint dilution assays. The pathogenic SIV challenge stocks used in these experiments were generated by expanding SIV_mac239X_ [51] in RM PBMCs and were titered using the CMMT-CD4-LTR-β-Gal sMAGI cell assay (National Institutes of Health AIDS Reagent Program).

### SIV detection assays

Plasma SIV RNA levels were determined using a gag-targeted quantitative real time/digital RT-PCR format assay, essentially as previously described, with 6 replicate reactions analyzed per extracted sample for assay thresholds of 15 SIV RNA copies/ml [7,52,53]. Quantitative assessment of SIV DNA and RNA in cells and tissues was performed using gag targeted, nested quantitative hybrid real-time/digital RT-PCR and PCR assays, as previously described [7,52,53]. SIV RNA or DNA copy numbers were normalized based on quantitation of a single copy rhesus genomic DNA sequence from the *CCR5* locus from the same specimen, as described, to allow normalization of SIV RNA or DNA copy numbers per 10^8^ diploid genome cell equivalents. Ten replicate reactions were performed with aliquots of extracted DNA or RNA from each sample, with two additional spiked internal control reactions performed with each sample to assess potential reaction inhibition. Samples that did not yield any positive results across the replicate reactions were reported as a value of “less than” the value that would apply for one positive reaction out of 10. Threshold sensitivities for individual specimens varied as a function of the number of cells or amount of tissue available and analyzed; for graphing consistency, values are plotted with a common nominal sensitivity threshold.

### Immunologic assays

SIV-specific CD4^+^ and CD8^+^ T cell responses were measured in peripheral blood mononuclear cells (PBMC) by flow cytometric intracellular cytokine analysis, as previously described [6–8]. Briefly, individual or whole protein mixes of sequential 15-mer peptides (11 amino acid overlap) spanning the SIV_mac239_ Gag, 5’-Pol, Nef, Rev, Tat, and Vif proteins or individual SIV_mac239_ Gag supertope peptides [Gag_211-222_ (53), Gag_276-284_ (69), Gag_290-301_ (73), Gag_482-490_ (120)] were used as antigens in conjunction with anti-CD28 (CD28.2, Purified 500 ng/test: eBioscience, Custom Bulk 7014-0289-M050) and anti-CD49d stimulatory mAb (9F10, Purified 500 ng/test: eBioscience, Custom Bulk 7014-0499-M050). Mononuclear cells were incubated at 37°C with individual peptides or peptide mixes and antibodies for 1h, followed by an additional 8h incubation in the presence of Brefeldin A (5 μg ml^−1^; Sigma-Aldrich). Stimulation in the absence of peptides served as background control. After incubation, stimulated cells were stored at 4°C until staining with combinations of fluorochrome-conjugated monoclonal antibodies including: anti-CD3 (SP34-2: Alexa700; BD Biosciences, Custom Bulk 624040, PerCP-Cy5.5; BD Biosciences, Custom Bulk 624060, and Pacific Blue; BD Biosciences, Custom Bulk 624034), anti-CD4 (L200: AmCyan; BD Biosciences, Custom Bulk 658025, BV510; BD Biosciences, Custom Bulk 624340 and BUV395; BD Biosciences, Custom Bulk 624165), anti-CD8a (SK1: PerCP-eFluor710; Life Tech, Custom Bulk CUST04424), anti-TNF-α (MAB11: FITC; Life Tech, Custom Bulk CUST03355 and PE; Life Tech, Custom Bulk CUST04596), anti-IFN-γ (B27: APC; BioLegend) and anti-CD69 (FN50: PE; eBioscience, Custom Bulk CUST01282 and PE/Dazzle594; BioLegend) and for polycytokine analyses, anti-IL-2 (MQ1-17H12; PE Cy-7; Biolegend), and anti-MIP-1β (D21-1351, BV421; BD Biosciences). For analysis of memory differentiation (central- vs transitional- vs effector-memory) of SIV Gag-specific CD4^+^ and CD8^+^ T cells, PBMC were stimulated as described above, except that the CD28 co-stimulatory mAb was used as a fluorochrome conjugate to allow CD28 expression levels to be later assessed by flow cytometry, and in these experiments, cells were surface-stained after incubation for lineage markers CD3, CD4, CD8, CD95 and CCR7 (see below for mAb clones) prior to fixation/permeabilization and then intracellular staining for response markers (CD69, IFN-γ, TNF-α; note that Brefeldin A treatment preserves the pre-stimulation cell-surface expression phenotype of phenotypic markers examined in this study).

Flow cytometry analysis was preformed using an LSR-II flow cytometer (BD Biosciences). Data analysis was performed using FlowJo software (Tree Star). In all analyses, gating on the lymphocyte population was followed by the separation of the CD3^+^ T cell subset and progressive gating on CD4^+^ and CD8^+^ T cell subsets. Antigen-responding cells in both CD4^+^ and CD8^+^ T cell populations were determined by their intracellular expression of CD69 and either or both of the cytokines IFN-γ and TNF-α (or in polycytokine analyses, expression of CD69 and any combination of the cytokines: IFN-γ, TNF-α, IL-2, MIP-1β). For longitudinal immunological assessment during vaccine and challenge phases, assay limit of detection was determined, as previously described [54], with 0.05% after background subtraction being the minimum threshold used in this study. After background subtraction, the raw response frequencies above the assay limit of detection were “memory-corrected” (e.g., % responding out of the memory population), as previously described [7,52–54], using combinations of the following fluorochrome-conjugated mAbs to define the memory vs naïve subsets: CD3 (SP34-2: Alexa700 and PerCP-Cy5.5), CD4 (L200: AmCyan and BV510), CD8a (SK-1: PerCP-eFluor710, RPA-T8: APC; BioLegend), TNF-α (MAB11; FITC), IFN-γ (B27; APC), CD69 (FN50; PE), CD28 (CD28.2; PE/Dazzle 594, BioLegend and BV510, BD Biosciences), CD95 (DX2; PE, BioLegend and PE-Cy7, BioLegend), CCR7 (15053; Biotin, R&D Systems), streptavidin (Pacific Blue, Life Tech and BV605; BD Biosciences, Custom Bulk 624342) and Ki67 (B56; FITC, BD Biosciences, Custom Bulk 624046). For memory phenotype analysis of SIV Gag-specific T cells, all CD4^+^ or CD8^+^ T cells expressing CD69 plus IFN-γ and/or TNF-α were first Boolean OR gated, and then this overall Ag-responding population was subdivided into the memory subsets of interest on the basis of surface phenotype (CCR7 vs CD28). Similarly, for polycytokine analysis of SIV Gag-specific T cells, all CD4^+^ or CD8^+^ T cells expressing CD69 plus cytokines were Boolean OR gated and polyfunctionality was delineated with any combination of the four cytokines tested (IFN-γ, TNF-α, IL-2, MIP-1β) using the Boolean AND function.

### RNA sequencing

Whole blood was collected from RMs in PAXgene RNA tubes (PreAnalytiX) following the manufacturer’s procedures. (PreAnalytiX). RNA was isolated using PAXgene Blood miRNA kits (Qiagen) following the protocol provided with the kit that included an on-column DNase treatment. The quality and concentration of the recovered RNA was determined using a LabChip GXII (PerkinElmer) instrument and a ribogreen-based RNA assay, respectively. mRNA-seq libraries were constructed using Illumina TruSeq Stranded mRNA HT kit following the manufacturer’s recommended protocol. Libraries were sequenced on an Illumina NextSeq500 sequencer using Illumina NextSeq 500/550 High Output v2 kits (150 cycles) following the manufacturer’s protocol for sample handling and loading. Sequencing run metrics were visualized for quality assurance using Illumina’s BaseSpace platform, and the quality of mRNA-seq reads were assessed using FastQC version 0.11.3 (http://www.bioinformatics.babraham.ac.uk/projects/fastqc). Both rhesus globin and ribosomal sequences were filtered via alignments with Bowtie v2.1.0 [55]. Adapters were digitally removed using cutadapt, version 1.8.3: https://doi.org/10.14806/ej.17.1.200. Subsequently, a minimum of twenty million raw reads were mapped to the *Macaque mulatta* genome Mmul_10 (obtained from iGenomes: https://support.illumina.com/sequencing/sequencing_software/igenome.html) with STAR v2.4.0h1 [56] followed by HTSeq-count v0.6.1p1 [57] to generate gene counts.

### Preparation for analysis of differential expression

Based on the raw read counts, outlier samples and genes with a maximum expression across all samples below 100 counts were removed. Using R (v 3.6.0)/Bioconductor(v 3.9), counts were then transformed into counts per million using the voom function [58] in the R library *limma* [59] with a smoothing window of 0.1. CPMs were normalized using the quantile method. Differential expression was performed using the *limma* package in R/Bioconductor [60]. Additional graphics packages were utilized for the visualization of numbers of DE genes (*ggplot2;* https://ggplot2.tidyverse.org) and heat maps (*gplots;* https://www.rdocumentation.org/packages/gplots/versions/3.1.0) using wk0, d0 as the common baseline comparator for each animal in the training set and the validation set cohorts. Heatmap data were transformed to Z scale (mean subtracted and divided by standard deviation) prior to display.

### Differential expression analysis

To determine the list of significantly differentially expressed genes in the RhCMV/SIV vector-vaccinated cohort as well as in the rRh-Het-IL-15 experiment, we used the *lm* function in the R library *stats*. Genes with a false discovery rate (FDR)-adjusted p-value ≤ 0.05 and absolute log_2_ fold-change (FC) (compared to baseline) above 1.5 were defined as significantly differentially expressed (DE). To define gene correlates of vaccine protection we determined the set of genes for which the baseline-subtracted expression values significantly differed between protected and non-protected outcome groups of the training set cohort (DDE). Genes with FDR-adjusted p≤0.05 and absolute log_2_(FC) (across protection groups) above log_2_ 1.5 were defined as significantly differentially DE (DDE). These genes were identified using the interaction effect between time point (compared to baseline) and vaccine protection.

### Principle component analysis (PCA)

PCA was performed on the per-timepoint mean log2(FC) values, using all expressed genes and averaged over the RMs within each treatment and outcome group. We used the R function PCA in the *FactoMineR* library, with variance scaling enabled and keeping 7 dimensions in the output. For each of the top 7 dimensions, we included the genes most highly correlated with the PC. The number of genes selected from each dimension was the dimension’s percentage explained variance times 2.5 (an arbitrary value selected to balance figure size with information content).

### Exploratory pathway analyses

Enrichment tests and network analyses for pathways and upstream regulators were performed using Ingenuity Pathway Analysis [61] (Apr 2021 version). IL-15-regulated networks were identified using GeneMANIA [62]. Co-expression analyses were performed only on DE genes. We conducted clustering analysis using Ward clustering and Euclidean distance on the union of log_2_(FC) values using the *WGCNA*, *heatmap*.*2*, and *EdgeR* Bioconductor packages in R [63–65].

### Hierarchical cluster analysis and heat map generation

For correlation analysis of the fold-change values displayed in heatmaps, we used midweight bicorrelation, a modified version of Pearson correlation (bicor function in the R library *WGCNA*), and complete linkage clustering. The clusters were defined using the cutree function (R library *stats*) [66]. The clustering of DDE genes (**Fig 3**) was cut at the height 1.4 which corresponds to a midweight bicorrelation coefficent of -0.4, because the analysis is constructed by shifting the correlations to the range (0,2). At this height, the genes were divided into three biologically distinct clusters with minimal loss of genes to the smaller clusters. In the clustering of IL-15-regulatead DDE genes (**Fig 4**) we shifted the cutoff to 1.3, as to divide both up- and down regulated genes into a larger cluster where DDE and IL-15 signatures were correlated, and a smaller cluster where DDE and IL-15 signatures were anti-correlated. The heatmaps were drawn using the heatmap.2 function (R library *gplots*).

### Permutation testing

To formally test whether the DDE and pathway-linked gene signatures significantly differentiate protected vs. unprotected RMs over the course of study, we devised and followed a formal statistical analysis plan. Briefly, we defined a test statistic aggregating over genes and time and compared this to a null distribution that controls for the observed data (including all correlations across genes, which is ignored in the primary linear modeling analysis described above). In this procedure we first calculate, for each gene, the absolute value of the mean over time of the difference in log_2_(FC) across protection groups. For the DDE analysis, the primary test statistic (“sum”) is the sum of the above value across all significantly DDE genes. We then compare this value to its empirical null distribution, approximated by sampling protection outcomes within each treatment group over 5000 permutations sampled with replacement. Note that for each permutation, the list of significantly DDE genes is allowed to change (the value of the test statistic was set to zero for permutations in which no genes were significantly DDE). We then repeated this analysis, where instead of the DDE gene list, we used the gene lists related to the IL-15 response, immune cell signaling pathway, TLR signaling pathway, and inflammasome/cell death pathway. These gene lists are fixed and do not vary across permutations; otherwise, all other aspects of the fixed-list analyses were identical to the DDE permutation analysis. Unadjusted p-values are the proportion of the 5000 permutations ≥ the observed test statistic (a one-sided test; the statistic is an absolute value, so it is always positive). To evaluate the hypothesis that the IL-15 response signature component of DDE genes is expressed among protected RMs more than non-protected RMs, we devised a “contrast” test statistic that directly addresses the hypothesis that protected RMs have a response to vaccination consistent with a response to IL-15 administration. This test statistic compares across protection categories a summary measure indicating the extent to which the gene response to RhCMV/SIV vaccination is consistent with the response to IL-15 administration (up-regulated genes going up, down-regulated genes going down), by measuring the difference across two gene lists of the sum of the average log_2_(FC) over time: the total among those genes up-regulated by IL-15 administration minus the total among those down-regulated by it. For these analyses, instead of sampling from all possible permutations with replacement, we evaluated the support of the null hypothesis exhaustively resulting in an exact test (with 5005 possible configurations in evaluating the validation cohort, and 6435 when evaluating the subQ cohort). Unadjusted p-values for the “sum” tests are the proportion of the null distribution ≥ the observed test statistic (testing a one-sided hypothesis), and unadjusted p-values for the “contrast” statistic are two-sided (computed as double the smallest one-sided p-value). For analyses of single gene DDE statistics (for the IL-15 gene), we repeated the main DDE fixed-list analyses on a list consisting of the single *IL-15* gene. For the primary “sum” analysis of baseline DE, we repeated the primary DDE analysis (in which DDE is redefined anew in each permutation) but employed baseline values instead of mean-over-time of the change-from-baseline values; as with the DDE analysis, this analysis leads to a zero-inflated null distribution since under many permutations there are no genes that are significantly differentially expressed.

### Predictor definition and evaluation

To formally evaluate linear predictors of binary challenge outcomes, we employed the following procedure. For the scalar, continuous-valued predictors defined by the IL15xDDE cluster A and cluster B contrast statistic sum (cluster A) –sum (cluster B) of the average change-from-baseline over time after baseline, we used the roc function of the *pROC* library in R to compute the area under the receiver operating characteristic curve (AUC-ROC) and we used the confusionMatrix function of the *caret* library to compute classification accuracy using the decision threshold optimized for predicting the protection outcome [67,68]. These statistics and threshold were computed as functions of the estimated probability of protection, which we computed by first employing logistic regression to model the outcome as a function of the respective contrast statistic.

### Additional statistical analyses

For fixed-list baseline DE analyses (**Fig 6A and 6B**) including the single-gene analysis, we compared normalized expression values at baseline across protection groups using two-sided two-sample Wilcoxon tests. Pre-vaccination transcriptome correlation analysis was conducted using Pearson correlation. Viral load and immunologic data are presented as boxplots with jittered points and a box from 1st to 3rd quartiles (IQR) and a line at the median, with whiskers extending to the farthest data point within 1.5×IQR above and below the box. Analyses of longitudinal ICS data were performed by calculating the per-RM average T-cell response over three periods: post-prime peak (2–6 weeks), post-boost peak (20–24 weeks), and plateau (61–90 weeks), and comparing these values between RMs receiving subQ vs. oral vaccine using the nonparametric Wilcoxon rank-sum test. All P*-*values are based on two-sided tests and unadjusted except where noted. Adjusted P*-*values were computed using the Holm procedure for family-wise error rate control.

Links to software:

STAR aligner: https://github.com/alexdobin/STAR

Bowtie2: http://bowtie-bio.sourceforge.net/bowtie2/index.shtml

HTseq: https://github.com/simon-anders/htseq

FASTQC: https://www.bioinformatics.babraham.ac.uk/projects/fastqc/

Cutadapt: https://github.com/marcelm/cutadapt

Venny: http://bioinfogp.cnb.csic.es/tools/venny/index.html

Ingenuity Pathway Analysis: https://digitalinsights.qiagen.com/products-overview/discovery-insights-portfolio/analysis-and-visualization/qiagen-ipa/

Genemania: https://genemania.org/

## Supporting information

S1 Table DE genes. Gene expression of SubQ and oral cohort animals was evaluated and is shown as fold-changes (from baseline). Data set includes unadjusted P-values, and adjusted p-values of DE genes of 68–1 oral and subQ cohort RMs vaccinated with RhCMV/SIV.
(XLSX)

S2 Table DDE genes. Data set includes all DDE genes, defined as the difference in DE gene expression across protection groups for 68–1 oral and subQ cohort RMs vaccinated with RhCMV/SIV, with unadjusted and adjusted p-values.
(XLSX)

S3 Table IL-15 response genes. Data set includes gene of SubQ and oral cohort fold-changes (from baseline), unadjusted p-values, and adjusted p-values of IL-15 response genes for all time points (Sheet 1) and 1day after rRh-Het-IL-15 administration (Sheet 2).
(XLSX)

S4 Table Overlap of DDE and IL-15 response genes. Data set includes genes from SubQ and oral cohort shown as fold-changes (from baseline) with unadjusted p-values, and adjusted p-values of DDE genes that are also IL-15 response genes.
(XLSX)

S5 Table Validation cohort DE genes. Data set includes all DE genes from the validation cohort, showing fold-changes (from baseline), unadjusted p-values, and adjusted p-values.
(XLSX)

S6 Table Overlap of DDE and IL-15 response genes in the validation cohort. Data set shows genes within the validation cohort that were DDE genes from 68–1 oral and subQ cohort RMs that are also IL-15 response genes. Data includes fold-changes, unadjusted p-values, and adjusted p-values for each gene.
(XLSX)

S7 Table Baseline IL-15 response gene Z scores. Data set includes Z score of each IL-15 response genes at baseline for each RM of the original subQ and oral cohorts.
(XLSX)

S1 Fig Immunogenicity of 68–1 RhCMV/SIV vectors in subcutaneously vs. orally vaccinated RMs. **A,B.** Longitudinal (A) and plateau-phase (B) analysis of the frequencies of vaccine-elicited, SIV Gag, Rev/Tat/Nef (RTN) and Pol insert-specific CD4^+^ and CD8^+^ T cell responses in the memory compartment of peripheral blood from study RMs. In A, the background-subtracted frequencies of CD4^+^ or CD8^+^ cells producing TNF and/or IFN-γ by flow cytometric ICS assay to overlapping 15mer peptide mixes comprising each of the SIV inserts within the memory subsets were summed for overall responses, with the figure showing the mean (+ SEM) of these overall responses at each time point for each RM cohort (oral vs. subQ). Boxplots in B compare the overall and individual SIV insert-specific CD4^+^ and CD8^+^ T cell response frequencies between the subQ and oral vaccine groups at the end of the vaccine phase (each data point is the mean of response frequencies in all samples from a given RM from wks 61–90 after first vaccination). Two-sided Wilcoxon rank-sum tests were used to compare the significance of differences in plateau-phase response frequencies between the vaccine groups (oral vs. subQ 68–1 RhCMV/SIV vectors). **C.** Longitudinal analysis of the magnitude of CD8^+^ T cell responses to SIV Gag supertopes in the peripheral blood memory compartment, determined by the same ICS assay described in panel A. Gag_211-222_ (53) and Gag_290-301_ (73) are MHC-II-restricted supertopes; Gag_276-284_ (69) and Gag_482-490_ (120) are MHC-E-restricted supertopes. **D.** Boxplots compare the memory differentiation phenotype of the vaccine-elicited CD4^+^ and CD8^+^ T cells in peripheral blood responding to SIV Gag peptide mix with TNF and/or IFN-γ, at or after 24 wks post-initial RhCMV/SIV vector vaccination. Memory differentiation state was assessed by CD28 and CCR7 expression, delineating central memory (T_CM_), transitional effector-memory (T_TrEM_), and effector-memory (T_EM_) subsets, as designated. Two-sided Wilcoxon rank-sum tests were used to compare the significance of differences in the fraction of responding cells with a T_CM_ phenotype (reciprocal of fraction with effector differentiation − T_TrEM_ + T_EM_). **(E)** Boxplots compare the frequency of RhCMV-elicited CD4^+^ and CD8^+^ T cells in PBMCs responding to SIV Gag peptides with TNF-α, IFN-γ, IL-2, and MIP-1β production, alone and in all combinations. The two-sided Wilcoxon rank sum test was used to pairwise compare differences between the fraction of SIV Gag-specific CD4^+^ and CD8^+^ T cells expressing one, two, three, or four cytokines. The n and p-values for all analyses are shown in the figure.
(EPS)

S2 Fig The magnitude and phenotype of SIV-specific CD4^+^ and CD8^+^ T cell responses in blood do not predict 68–1 RhCMV/SIV vector efficacy. **A,B**. Longitudinal (A) and plateau-phase (B) comparison of the frequencies of vaccine-elicited, SIV Gag, Rev/Tat/Nef (RTN) and Pol insert-specific CD4^+^ and CD8^+^ T cell responses in peripheral blood (as described in **S1 Fig**) in RMs from both vaccinated cohorts (subQ and oral) that were protected (red) vs. non-protected (black) after challenge. **C.** Longitudinal analysis of the magnitude of CD8^+^ T cell responses to SIV Gag supertopes in the peripheral blood memory compartment (as described in **S1 Fig**) in RMs from both vaccinated cohorts that were protected vs. non-protected after challenge. **D,E.** Boxplots compare the memory differentiation of (D), and the relative proportion of TNF-α, IFN-γ, IL-2, and MIP-1-β production within (E) vaccine-elicited, SIVgag-specific CD4^+^ and CD8^+^ memory T cells in late vaccine-phase peripheral blood (as described in **S1 Fig**) of RMs from both vaccinated cohorts that were protected vs. non-protected after challenge. Statistical analysis of data sets comparing protected vs. non-protected RMs was performed as described in **S1 Fig**, with the n for each analysis and the p-values shown in the figure.
(EPS)

S3 Fig Outcome prediction based on IL-15xDDE Cluster A—Cluster B Contrast. Y axis shows predicted probability of protection based on a simple logistic regression model fit to the average change-from-baseline contrast statistic for each animal, sorted by rank (oral group RMs as squares, subQ group RMs as circles; protected in red). Accuracy is shown for each group separately and for the groups together. It is based on the decision threshold optimized for the two groups together (shown as a horizontal line; animals above this line are predicted as protected). Area under the receiver operating characteristic curve (AUC-ROC) at this optimal threshold and over the whole curve are both reported on the figure, indicating respectable, but imperfect classification based on this simple contrast statistic.
(EPS)

## Abbreviations

Bo: boost
DE: differentially expressed
DDE: differential expression of DE genes (protected vs. non-protected), EM, effector-memory
FC: fold-change
FDR: false discovery rate
ICS: intracellular cytokine staining
IRF: interferon regulatory factor
ISGs: interferon stimulated genes
ONPRC: Oregon National Primate Research Center
PI3K: phosphatidylinositol responsive kinase
Pr: prime
preCh: pre-challenge
PC: principal component
PCA: principal component analysis
RhCMV: Rhesus Cytomegalovirus
RMs: rhesus macaques
SIV: Simian Immunodeficiency Virus
subQ: subcutaneous
