# Supplementary material for: Interleukin-15 response signature predicts RhCMV/SIV vaccine efficacy
Source: PLoS Pathog. 2021 Jul 6;17(7):e1009278. doi: 10.1371/journal.ppat.1009278 (PMC8284654; doi:10.1371/journal.ppat.1009278)
Supplement: Attachment — Submitted filename: Response to Reviewer comments-final.docx [file ppat.1009278.s011.docx]

**PPATHOGENS-D-21-00034**

**Response to Reviewer comments**

We sincerely thank the reviewers for their time and effort to review our manuscript. The comments were very helpful, resulting in a significantly improved manuscript.

We have responded fully to all comments via 1) text revision, 2) re-mapping all sequence read data to the MM10 genome, 3) re-running full and extended statistics on the differential gene expression output from the MM10 genome mapping of reads, 4) completely rebuilding Figures 2-5 based on these new analyses, and 5) adding a deep analysis of the baseline gene expression signature now presented as a new figure (Figure 6), all as requested by the reviewers.

We note that from these new analyses we now reveal that the vaccine-induced IL-15 signature observed in protected animals in our original subQ and oral vaccine cohorts is fully and significantly captured by our validation cohort, confirming the correlation of this signature with vaccine protection.

Moreover, we have conducted a deep examination of the baseline signature for any presence of the *protective* gene expression signature prior to vaccination (as suggested by Reviewer). We found that monkeys that were ***not*** protected by the vaccine manifested significantly higher baseline blood IL-15 response gene expression compared to those monkeys that were actually protected by the vaccine. In other words, monkeys that manifested the post-vaccination enhancement of IL-15 signaling (change-from-baseline) and were subsequently protected from challenge tended to have a lower (more quiescent) IL-15 signaling pathway at baseline. Biologically, this profile of response and outcome captures the relationship of cytokine-induced gene expression and outcomes that we previously described as “innate immune tolerance” in our studies of type I interferon therapy in HCV-infected human cohorts wherein basal interferon and interferon-induced gene expression forces the cell into a state of tolerance to the actions of the cytokine/interferon. We now present these data as Figure 6 and have added new text in the results and discussion to describe our analysis approach, outcome, and how the concept of "cytokine tolerance" may be impacting the outcome of vaccination and virus challenge.

Details of these revisions follow each reviewer comment *in italic font*, shown below.

**REVIEWER 1**

1. Reviewer states that "Nevertheless, the data shown in Figure 5 are not compelling, and there is no statistical analysis associated with these results".

*Response: The application of rigorous standards for computation and presentation of p-values is indeed important to us and to the progress of science. The statistical analyses of these data were carefully devised and pre-specified, and in our originally presented analysis of the validation Rhesus Macaque (RM) cohort, the result of evaluating our pre-specified "contrast statistic" showed a p-value of 0.07. This statistic was not optimized in any way and was evaluated once. It employs the day 1 IL-15 response gene list from the independent experiment of IL-15 treatment described in our study. The statistic contrasts the gene expression of those genes that went up in response to IL-15 administration to those genes that went down, after removing any genes not found to be DDE (differential of the differentially expressed) genes in the subQ and oral data set. While it was a disappointing that this initial analysis did not reach the p = 0.05 level of significance, it was statistically sound, and we felt it supported our conclusions despite not quite reaching the significance threshold. We also felt that a more refined gene list would likely have improved the result. In response to the reviewer comments we have now reanalyzed the data with the updated MM10 genome. Note that we did not alter this statistical test in any way, and again conducted it once. This gene signature is statistically significant in the reanalysis (P=0.036). This is a very strong statistically supported result because of this clean rerun of the pre-specified statistical analysis plan. This clean, prespecified contrast statistic is also significant in the subQ group, evaluated by itself (P=0.041). While our prespecified plan did not originally include employing this test on the subQ and oral cohort data together, we have now performed this analysis and the result is highly significant (P<0.001). These analyses collectively provide solid statistical support that the genes found to be differentially expressed (DE) in response to IL-15 administration are also differentially changed in response to vaccination among protected animals in both the training (subQ and oral) and validation cohorts.*

2. Can protection be predicted prior to challenge?

*Response: First, we would emphasize that the goal of the reported studies was to use transcriptomic analysis to determine an immunologic basis for the highly reproducible 50-60% “all-or-nothing” efficacy of the 68-1 RhCMV/SIV vaccine. We sought to identify immunologic pathways that significantly correlated with outcome, and as mentioned above, we succeeded in this goal, demonstrating that a gene expression signature corresponding to IL-15 signaling significantly correlated with efficacy in both a training and a validation cohort. The identification of such a strong correlate of vaccine efficacy is unusual in any vaccine setting. For example, this correlate is stronger than the putative correlates of efficacy identified for the HIV vaccine studied in the RV144 vaccine trial. Going beyond this overall correlation to identify a signature that could accurately predict challenge outcome in individual vaccinated monkeys is a considerably more difficult task, especially using the relatively small n’s we worked with in this study. While we could not hope to have already achieved perfect individual-level prediction after studying so few animals with transcriptomics, the signature that we identified is a good start toward a predictor of individual-level outcomes, showing that the overall predictive value of the IL-15 signaling status before and after vaccination is 77% (ROC AUC 0.78) for the oral and subQ* chorts *(see last paragraph of results section and* ***Revised*** ***Figs. 6 and S3****). Interestingly, at both high and low ends of the IL-15 signaling spectrum, the correlation with outcome is nearly perfect, with less accurate prediction in the middle – the intermediate range of IL-15 signaling. We would also note that this new figure clarifies the IL-15 signaling overlap between protected and non-protected animals, responding to the reviewer’s comment. Finally, we would note that while the overall individual monkey prediction capability is not perfect, for an otherwise unoptimized, scalar predictor it performs rather well and we are highly encouraged that predictors can be optimized in the future with more challenge data, which will allow us to refine genes lists representing identified pathways, identify additional contributory pathways, and/or combine transcriptomic signaling parameters with parameters related to separately measured characteristics of the MHC-E-restricted CD8^+^ T cell response (see below).*

3. Experimental evidence that alteration of the IL-15 response (augmentation or ablation) either at the time of vaccination or challenge changes vaccine protectiveness would provide more compelling evidence that IL-15 is central to vaccine protection.

*Response: We do not disagree with the reviewer’s assertion that interventional studies will be necessary to unequivocally confirm direct involvement of IL-15 signaling in 68-1 RhCMV/SIV vaccine efficacy. However, we strongly argue that such interventional studies are far beyond the scope of the current study and manuscript. The Reviewer certainly appreciates that this is not a mouse model in which appropriately powered experiments using knock outs and/or other approaches could potentially be accomplished in a 2-3 months. The RM challenge studies described in our paper take a minimum of 2 years to complete and given that the baseline efficacy of our vaccine is ~55%, would require enormous group sizes to provide sufficient statistical power to conclusively demonstrate that IL-15 ablation alters vaccine efficacy from the 50% baseline. Indeed, our calculations, conducted using an asymptotic approximation of a two-sided Fisher's exact test with a 5% p-value threshold (alpha), indicate that for 80% power to distinguish a 55% from a 25% vaccine efficacy, we would need n = 100 RM total, n = 50 each in two groups! This sample size would also give 80% power to distinguish a 45% from a 75% efficacious vaccine. Such an experiment would cost many millions of $s and thus is clearly not feasible until we can more accurately target the protective mechanism(s) so as to plan for a much bigger effect size.*

*To emphasize the limitations of available putative interventions, we would point out, as shown in the paper, that administration of IL-15 at pharmacologic doses induces an immediate up-regulation of IL-15 signaling followed very quickly by a homeostatic counter-regulation that ablates that up-regulation and subsequently suppresses the IL-15 pathway for weeks. This regulation is quite different than the persistent upregulation of IL-15 signaling induced by 68-1 RhCMV/SIV vectors in subsequently protected monkeys. Indeed, contrary to first impression, it is, in our opinion, most likely that due to this counter signaling (cytokine response tolerance), such IL-15 administration therapy would reduce/ablate efficacy rather than augment efficacy. The newly added Figure 6 data to the manuscript showing that baseline IL-15 signaling tends to be higher in non-protected RM is consistent with this view. In addition, we don’t know when the IL-15 signaling contributes to protection, so timing of intervention would be guess-work and at present we have no validated technology that would provide a constant low-level triggering of IL-15 signaling that would have the potential to better mimic the signaling pattern in 68-1 RhCMV/SIV protected RM.*

*Moreover, we note that ablating IL-15 activity in monkeys is equally fraught with complexity and uncertainty. Dr. Picker is the expert on this, having developed and extensively tested a rhesusized anti-IL-15 monoclonal antibody (mAb) in RM (J. Immunol, 197:1183-98, 2016; J. Immunol., 203:2928-2943, 2019). As shown in these papers, the issue is that this mAb-mediated IL-15 ablation results in enormous changes to the immune system, including complete loss of all NK cells, reduction in tissue-based memory T cells and induction a dramatic high turnover state for both CD4^+^ and CD8^+^ memory T cells. Given these extensive generalized changes, any effect of such anti-IL-15 mAb therapy on 68-1 RhCMV/SIV efficacy would be extremely difficult to interpret. Whether such IL-15 neutralization could be “toned down” to simply take the edge off excess IL-15 signaling is an open question, but such an intervention does not exist at present. Publication of this paper, convincingly showing a correlation between IL-15 signaling and 68-1 RhCMV/SIV efficacy, would provide our group and potentially others the needed rationale to pursue the information needed to plan, justify and fund such mechanism-confirming interventional studies, but it would not be possible to provide such evidence in this manuscript. Strong statistical correlation data, such as presented in this paper, are a well-used and highly successful initial step in the delineation of biologic mechanisms.*

4. Identification of definitive parameters that distinguish protected versus unprotected animals prior to challenge would strengthen the argument that a true signature exists.

*Response: This point is very similar to this reviewer’s point #2, responded to above. Again, we would emphasize that the activity of IL-15-associated gene pathway described in the paper during the vaccine phase does indeed significantly correlate with outcome after challenge (vaccine efficacy) across 3 RM cohorts. We have now even shown that the baseline activity of the IL-15 signaling genes also significantly correlate. These data, and as indicated below, the linkage between MHC-E-restricted CD8^+^ T cell responses, provides two key insights into the unique mechanism(s) underlying the unprecedented “replication arrest” efficacy of 68-1 RhCMV/SIV vectors. Indeed, given the lack of precise understanding of how most licensed vaccines mediate efficacy, we argue that identification of both MHC-E-restricted CD8 T cell responses and this innate immune state defined by IL-15 response genes as required for RhCMV-SIV protection are among the clearest immune correlates of vaccine protection yet defined, which is remarkable given the unique nature of 68-1 RhCMV/SIV vaccine protection.*

*We have not (at least not yet!) fully characterized the relationship between the requirement for both MHC-E-restricted CD8s and up-regulation of IL-15 signaling during vaccine phase, and we would posit that the nature of this interaction and the basis for differing IL-15 response profiles among animals will be key to identifying even more accurate parameters to predict challenge outcome in individual vaccinated monkeys (see below). However, we hope that the reviewer would agree that such characterization is well beyond the scope of this paper.*

**REVIEWER 2**

1. Given that extensive prior work has established significant portion of the mechanism by which this vaccine affords protection (non-conventional MHC restriction) – the impact of the current study is lessened.

*Response: We respectfully, but strongly disagree with this assertion. Indeed, we argue just the opposite. The reviewer is correct that both published (Science, 372: eabe9233, 2021; Science Immunol, 6: eabg5413, 2021) and soon-to-be-published data convincingly link SIV-specific, MHC-E-restricted CD8^+^ T cells to 68-1 RhCMV/SIV vaccine efficacy and strongly support the hypothesis that SIV-specific, MHC-E-restricted CD8^+^ T cells are the adaptive immune response that mediates vaccine protection (see response-to-review* ***Fig. 1****). RhCMV/SIV vector-elicited MHC-Ia and MHC-II-restricted CD8^+^ T cells, though similar in character to MHC-E-restricted CD8^+^ T cells, are not efficacious. However, even when 68-1 RhCMV/SIV vectors are configured to elicit only MHC-E-restricted CD8^+^ T cell responses (miR-126 restricted 68-1 RhCMV/SIV vectors, manuscript-in-preparation), the rate of efficacy is still only 57%, not different from the parent 68-1 RhCMV/SIV vectors that show mixed MHC-E- and MHC-II-restricted responses (response-to-review* ***Fig. 1****); moreover, the efficacy of these MHC-E-only vectors is not predicted by the magnitude of the MHC-E-restricted CD8^+^ T cell response in blood (data not shown). Thus, although MHC-E-restricted CD8^+^ T cells appear to be required for protection, they are not, by themselves, sufficient for protection. Something else is needed. The validated efficacy-predictive transcriptomic signature identified in this paper points to the “something else” being an independent innate immune/immunoregulatory response to vaccination that enables the SIV-specific responses to achieve protection. Given the prominence of IL-15 signaling in this signature we submit that the most likely explanation, as discussed in the manuscript, is that the IL-15 signaling is necessary to functionally tune the MHC-E-restricted, SIV-specific CD8^+^ T cells for efficacy (although we do not rule out contributions by other cell types such as NK cells). Obviously, the specific nature of this putative tuning mechanism is a great interest and will be the focus of our future research, but even at this point, it is clear from our results that efficacy can only be explained by consideration of both the association of efficacy with MHC-E-restricted CD8^+^ T cells and the transcriptomic signature reported in this paper. Therefore, as mentioned above, the results of this study are just as important for understanding 68-1 RhCMV/SIV efficacy as the studies linking this efficacy to MHC-E-restricted CD8^+^ T cell responses. The revised manuscript discussion goes into considerable detail on this point.*

2. The current study conclusively associates a gene signature with protection but offers little insight into how this signature mechanistically acts. However, these concerns are mitigated by the clear importance of the scientific problem (understanding all aspects how this promising vaccine platform works). Lastly- in my opinion, this study is an example of an effective transcriptomics study – it has provided a high confidence hypothesis to explain a complex biomedical question, i.e. the association of IL-15 signaling to RhCMV-mediated protection- and while further validation (i.e. targeting IL-15) will be critical to the advancement of this model – they are beyond the scope of the current study.

*Response: We greatly appreciate the reviewer’s comments on the effectiveness of our study, an agreement that is reflected in our very similar responses to Reviewer #1. We would also agree that we now have key insights that will guide future studies aimed at defining the molecular mechanisms of immune programming for 68-1 RhCMV/SIV vaccine protection.*

3. The method in which the data presented in Figure 2D is somewhat geared towards presenting coherent heat maps: by using genes in PC1 together they will naturally act the same way. While it's fine to leave this figure – I would suggest augmenting Figure 2 with a more straightforward traditional analysis: (i) provide Boolean diagrams of the overlaps of DEGs between the Oral and SubQ protected gene expression, and also with the unprotected. (ii) Include a heat map in which the top ~1000-2000 DEGs across all comparisons are clustered – to show natural coherence of the genes (i.e., without the filtering effect of the PCs).

*Response: There seems to be a misunderstanding here. The linear DE and DDE analyses were independent of the PCA – no filtering was performed. The similarity between the DDE signature, and PC1 was entirely a downstream discovery. We have added new text to make this clear in the revised paper.*

*To respond to the reviewer, we created Venn diagrams of the differentially expressed genes to complement the Fig. 2 heat map. At right here (response-to-review* ***Fig. 2****), we show that at W0D3, the oral group had a higher number of DE genes, and the DE genes in the subQ group were essentially a subgroup thereof. At W18D3, the protected subQ group showed a substantially higher number of DE genes than the oral. Finally, 88 weeks after prime both groups have moderate number of DE genes from baseline, with a substantial overlap. Strikingly, across the time series the overlap is larger in the protected group than the unprotected, supporting the existence of a protective signature. We feel that these Venn diagrams do not add new or useful information to the manuscript and therefore they are not included in the revised paper.*

**Fig. 2**

*As described above, the heat map of the protective signature includes all the genes significantly associated with vaccine protection based on linear modeling (DDE analysis, Fig. 3). We have also created a similar heat map including the 2000 genes as suggested by the reviewer, with the strongest p-values for any time point and group from the Figure 2 DE analysis. As shown in the heat map below (response-to-review* ***Fig. 3)****, similar to the Figure 3 DDE genes, this gene set shows a trend towards similar vaccine responses in all animals, with an enhanced response in the protected group. We feel that this heat map does not add useful or new information to the study, so we have not included it in the revised paper.*

*In our experience, it’s common for one particular signature to dominate even complex datasets such as this. PCA-informed heat maps, such as shown in Fig. 2, effectively lets the reader look past dominant trends and study otherwise hidden trends in the data. Therefore, we prefer to show the Fig. 2 DE heat map in its current form.*

**Fig. 3**

4. Figures 2A and 2B can be omitted, they don’t add much to the story, and given the way they are calculated, are not really insightful. Alternatively, cluster and/or heat maps of correlation co-efficient for post-vaccine time points should be shown for comparison.

*Response: Figs. 2A and 2B have been superseded by more definitive baseline analysis (new* ***Fig. 6****) and therefore have been removed. However, we did retain the PCA plot (Fig. 2C) showing the initial clustering of the data that suggested that these data segregate by protection status. We retained the PCA plot because we feel it provides some value for data interpretation. In our reanalysis, the data remain segregated by protection in the first two principal components, and this outcome provides some insight into the relevant genes contributing to protection. Together with the heat map in this and other figures, these panels show the insights from our investigation, and are relevant to understanding our findings.*

5. The rhesus annotation MMul_1 – is not widely used… do they mean MMUL_10? In any case, it would not be practical to redo the analysis, but further description of this annotation in terms of #’s of genes would be helpful in the Methods.

*Response: We used MMUL-1 in the previously reviewed version of the figure set because we had started the analyses with that genome version at the very beginning of this study. However, as stated above in the opening paragraph, we decided to take this opportunity to completely redo all the analyses using the Mmul_10 genome, thus mapping all sequencing reads to the latest version of the RM genome, reasoning (correctly it turned out) that the updated genome would refine our analysis. This full reanalysis included a full statistical re-analysis, and thus took us 2.5 months to complete our revisions. The end product, however, is striking – the data are improved due to the improved annotation of the MMUL-10 genome such that the significance of the identified gene expression signatures linked with vaccine protection is improved across the data set. Most importantly, the analyses of the IL-15/DDE overlap signature that linked with protection in the original subQ and oral RM cohorts is now significantly linked with protection of the validation cohort animals (Figure 5; see response to Reviewer #1 point #1). We therefore greatly appreciate the reviewer mentioning this issue, as addressing it substantially improved our analysis.*

6. The numbers of DEGs at some time-points seems abnormally high… according to the methods, the reference yielded ~12,000 transcripts for analysis, and the Oral Protected group had >2000 genes upregulated and >2000 genes downregulated for a total of >4000 genes of 12,000. Further explanation is warranted.

*Response: While we agree that the number of DE genes is high, we have observed similar number before in nonhuman primates undergoing vaccination or infection (e.g., in Barrenas, et al. Nature Communications, 2019). In general, 3-7 days after viral challenge, blood transcriptomes can show perturbations in thousands of genes. Over time, animals tend to stabilize at a new steady state with a moderate number of DE genes compared to baseline. Biologically speaking, a bulk blood transcriptome represents a complex mix of distinctly different cell types (monocytes, neutrophils, T cells, etc.). Increased abundance of only a single cell type could perturb the expression of a substantial number of genes. We have similar comment on DE gene numbers in the revised discussion.*

7. The Discussion is underdeveloped – likely from formatting the length for other journals. More in depth discussion of the caveats of the study, and potential mechanisms of IL15 beyond “non-conventional T cell responses” would be helpful. What about NK cells? Does IL15 enhance mucosal T cell responses?

*Response: We agree with the Reviewer and have considerably expanded the discussion to present concepts on mechanisms of IL-15 response, T cell response, and other gene network correlates of protection and their possible impact on mediating vaccine protection. For the reviewer’s information, we would comment that as yet unpublished data shows that 68-1 RhCMV/SIV vaccine efficacy is abrogated by anti-CD8β-mediated depletion prior to and during challenge. Since this treatment depletes CD8^+^ T cells but not NK cells, we conclude that the MHC-E-restricted CD8^+^ T cells are the primary adaptive component of protection, but, of course, this does not rule out a different role for NK cells, which are also strongly regulated by IL-15. The revised discussion does address the possibility that IL-15 signaling may contribute to outcome via actions on IL-15-responsive, non-CD8+ T cell types, such as NK cells and CD4+ effector-memory T cells. With regard to the question of enhanced mucosal T cell responses in our protected animals, we would first comment that we do not have data that directly addresses this question. Moreover, we have previously documented that SIV replication arrest mediated by 68-1 RhCMV/SIV vaccination after mucosal SIV challenge occurs in both mucosal sites (e.g., colon mucosa) and non-mucosal sites of early viral spread, including spleen, liver and certain lymph nodes (see ref #s 7 and 8). Thus, while mucosal immunity certainly contributes to vaccine efficacy, it cannot by itself explain efficacy, which also must depend on immunity in non-mucosal sites. We would therefore posit that the proposed beneficial effect of IL-15 signaling on efficacy is very likely systemic.*

8. Some minor typos, and potential corrections: is it PAXgene miRNA or RNA kits? In the Abstract “toll-lie”.

*Response: We have fixed these typos.*
