# Supplementary material for: Interleukin-15 response signature predicts RhCMV/SIV vaccine efficacy
Source: PLoS Pathog. 2021 Jul 6;17(7):e1009278. doi: 10.1371/journal.ppat.1009278 (PMC8284654; doi:10.1371/journal.ppat.1009278)
Supplement: Peer review history

# Peer review: Decision Letter 0

**DOI:** 10.1371/journal.ppat.1009278.r001

**Authors:** Guido Silvestri, Thomas J. Hope

5 Mar 2021

Dear Professor Gale Jr.,

Thank you very much for submitting your manuscript "Sustained IL-15 response signature predicts RhCMV/SIV vaccine efficacy" for consideration at PLOS Pathogens. As with all papers reviewed by the journal, your manuscript was reviewed by members of the editorial board and by two independent reviewers. In light of the reviews (below this email), we would like to invite the resubmission of a significantly-revised version that takes into account the reviewers' comments.

We cannot make any decision about publication until we have seen the revised manuscript and your response to the reviewers' comments. Your revised manuscript is also likely to be sent to reviewers for further evaluation.

When you are ready to resubmit, please upload the following:

[1] A letter containing a detailed list of your responses to the review comments and a description of the changes you have made in the manuscript. Please note while forming your response, if your article is accepted, you may have the opportunity to make the peer review history publicly available. The record will include editor decision letters (with reviews) and your responses to reviewer comments. If eligible, we will contact you to opt in or out.

[2] Two versions of the revised manuscript: one with either highlights or tracked changes denoting where the text has been changed; the other a clean version (uploaded as the manuscript file).

Important additional instructions are given below your reviewer comments.

Please prepare and submit your revised manuscript within 60 days. If you anticipate any delay, please let us know the expected resubmission date by replying to this email. Please note that revised manuscripts received after the 60-day due date may require evaluation and peer review similar to newly submitted manuscripts.

Thank you again for your submission. We hope that our editorial process has been constructive so far, and we welcome your feedback at any time. Please don't hesitate to contact us if you have any questions or comments.

Sincerely,

Guido Silvestri

Associate Editor

PLOS Pathogens

Thomas Hope

Section Editor

PLOS Pathogens

Kasturi Haldar

Editor-in-Chief

PLOS Pathogens

​orcid.org/0000-0001-5065-158X

Michael Malim

Editor-in-Chief

PLOS Pathogens

orcid.org/0000-0002-7699-2064

***********************

Reviewer's Responses to Questions

**Part I - Summary**

Please use this section to discuss strengths/weaknesses of study, novelty/significance, general execution and scholarship.

Reviewer #1: Although a novel RhCMV/SIV vaccine (68.1) has been demonstrated to provide virologic control and ultimately clearance of SIV infection in approximately half of vaccinated rhesus macaques, mechanisms that underlie this protection are not understood. This is an issue of great importance to the field as the correlates of immune protection from SIV and HIV are not understood. To attempt to provide mechanistic insight into these observations, transcriptomic analyses were performed in two cohorts of rhesus macaques who received the vaccine (n=30 and n=15) as well as five rhesus macaques who were treated with an IL-15 superagonist. The large number of animals studied is a strength of the study. This approach to understand mechanisms underlying protection conferred by the RhCMV/SIV vaccine is ambitious, but well reasoned.

Global transcriptomic profiling of mRNA in whole blood samples was performed at multiple time points and analyzed by principal component analysis and determination of differential expression of genes following vaccination. Differentially induced genes occurred rapidly after the first vaccine (day 1), were variably reduced, rebounded after the booster vaccine and were generally higher after the booster in the protected macaques compared to unprotected ones. Differences in this signature persisted and existed at the time of challenge. Further analysis of genes that were differentially expressed between protected and nonprotected animals revealed 2,272 genes that fell into four major clusters identified by clustering analysis. Vaccine induced gene clusters were associated with innate immune pathways including TLR signaling, cytokine response pathways, inflammasome/cell death signaling, and T cell/TCR signaling genes. IL-15 expression was enriched in the protected animals and was identified as a significant upstream regulator of the vaccine protection signature. Further evidence that the signature transcriptome was heavily influenced by IL-15 came from transcriptomic analyses in the animals treated with the IL-15 superagonist, who demonstrated at day 1 multiple transcriptome clusters that overlapped with those seen in the transcriptome signature associated with vaccine protection. These findings strengthen the conclusion that IL-15 is associated with many of the signature changes.

The whole blood gene signature was next validated using a different cohort of RM that had been vaccinated with both the 68-1 RhCMV/SIV vaccine and a non-protective variant (68-1.2). Six of the 15 vaccinated rhesus macaques exhibited the protected phenotype. Vaccine responses in both the original cohort and the validation cohort reportedly showed a higher correlation with the IL-15 response than in non-protected animals. Nevertheless, the data shown in Figure 5 are not compelling, and there is no statistical analysis associated with these results.

The authors conclude that the IL-15 response signature is a predictor of vaccine response that is linked to protection. They postulate that “tuning” by persistent induction of IL-15 and associated immunoregulatory pathways may be required for the MHC-E restricted SIV-specific CD8+ T cells to mediate arrest of viral replication. Because cells become refractory to IL-15 after continuous stimulation, they speculate that low, persistent IL-15 production is essential. They acknowledge that the basis for differential induction and maintenance of the protective signature among rhesus macaque is not clear, nor is the mechanism that accounts for RhCMV/SIV vectors to induce this response that is not induced by other vaccines.

The conclusions of the authors that the IL-15 response signature is a predictor of vaccine response is somewhat supported by the data. Nevertheless, it is not clear how much overlap there is between protected and unprotected animals. Can protection be predicted prior to challenge? The findings that are presented certainly suggest a potential role for IL-15. This information is interesting to the field, since IL-15 is under intensive investigation in several HIV cure protocols. Nevertheless, the studies presented in the manuscript are essentially descriptive, and not definitive. More compelling evidence that IL-15 is a key mediator of the vaccine-induced protection could come from attempts to modify the IL-15 response at the time of vaccination or challenge.

Reviewer #2: This study by Barrenas and colleagues investigates the mechanism of vaccine elicited protection from mucosal SIV challenge using the Rh-CMV vaccine in rhesus macaques. The RhCMV vaccine has been one of the most promising vaccine candidates in recent years, and a series of high impact papers have demonstrated its reproducibility and rigour in affording protection at slightly over 50% of challenged. In a set of follow-up studies, partial explanation of the protection has been attributed to the ability of the RhCMV vector to elicit long-standing mucosal SIV-specific effector memory CD8+ T cells, and to engender noncanonical restriction of CD8 T cells to recognize SIV antigens via MHC class II and class E presentation. In the current study, which has been highly anticipated, the authors employ high-throughput transcriptomics to query longitudinal gene expression after RhCMV vaccination. The goal was to identify additional pathways influenced by RhCMV 68-1 vaccine-elicited protection.

The study is comprised of a massive amount of RNA-Seq from a set of frequent longitudinal blood samples. The study is comprised of an impressive amount of complex biofinformatics, which required significant unorthodox statistical methodology. The study is very well-powered, and uses a very innovative study design: they leverage the well-powered nature of the parent NHP studies (n = 15 total) and the sample sizes of the subgroups of animals in matched sets of “protected” and “unproctected” with each group consisting of n = 7-9, more than adequate for robust power in a blood-based transcriptomics study expected effect sizes by most vaccines. The primary finding of the study is that animals exhibiting protection have a consistent and strong induction of IL15 signalling pathways. Finally – the study uses an exceptionally extensive orthologous validation of the IL15 gene signatures to support their model. Some of the weaknesses are 1. Given that extensive prior work has established significant portion of the mechanism by which this vaccine affords protection (non-conventional MHC restriction) – the impact of the current study is lessened 2. The current conclusively associates a gene signature with protection, but offers little insight into how this signature mechanistically acts. However, these concerns are mitigated by the clear importance of the scientific problem (understanding all aspects how this promising vaccine platform works). Lastly- in my opinion, this study is an example of an effective transcriptomics study – it has provided a high confidence hypothesis to explain a complex biomedical question, i.e. the association of IL15 signaling to RhCMV-mediated protection- and while further validation (i.e. targeting IL15) will be critical to the advancement of this model – they are beyond the scope of the current study.

**********

**Part II – Major Issues: Key Experiments Required for Acceptance**

Please use this section to detail the key new experiments or modifications of existing experiments that should be absolutely required to validate study conclusions.

Generally, there should be no more than 3 such required experiments or major modifications for a "Major Revision" recommendation. If more than 3 experiments are necessary to validate the study conclusions, then you are encouraged to recommend "Reject".

Reviewer #1: 1. Experimental evidence that alteration of the IL-15 response (augmentation or ablation) either at the time of vaccination or challenge changes vaccine protectiveness would provide more compelling evidence that IL-15 is central to vaccine protection.

2. Identification of definitive parameters that distinguish protected versus unprotected animals prior to challenge would strengthen the argument that a true signature exists.

Reviewer #2: 1. The method in which the data presented in Figure 2D is somewhat geared towards presenting coherent heatmaps: by using genes in PC1 together they will naturally act the same way. While its fine to leave this figure – I would suggest augmenting Figure 2 with a more straightforward traditional analysis: (i) provide Boolean diagrams of the overlaps of DEGs between the Oral and SubQ protected gene expression, and also with the unprotected. (ii) Include a heatmap in which the top ~1000-2000 DEGs across all comparisons are clustered – to show natural coherence of the genes (i.e. without the filtering effect of the PCs).

2. Figures 2A and 2B can be omitted, they don’t add much to the story, and given the way they are calculated, are not really insightful. Alternatively, cluster and/or heatmaps of correlation co-efficients for post-vaccine timepoints should be shown for comparison.

3. The rhesus annotation MMul_1 – is not widely used… do they mean Mmul_10? In any case, it would not be practical to redo the analysis, but further description of this annotation in terms of #’s of genes would be helpful in the Methods.

4. The numbers of DEGs at some time-points seems abnormally high… according to the methods, the reference yielded ~12,000 transcripts for analysis, and the Oral Protected group had >2000 genes upregulated and >2000 genes downregulated for a total of >4000 genes of 12,000. Further explanation is warranted.

5. The Discussion is underdeveloped – likely from formatting the length for other journals. More in depth discussion of the caveats of the study, and potential mechanisms of IL15 beyond “non-conventional T cell responses” would be helpful. What about NK cells? Does IL15 enhance mucosal T cell responses.

**********

**Part III – Minor Issues: Editorial and Data Presentation Modifications**

Please use this section for editorial suggestions as well as relatively minor modifications of existing data that would enhance clarity.

Reviewer #1: (No Response)

Reviewer #2: 1. Some minor typos, and potential corrections: is it PAXgene miRNA or RNA kits? In the Abstract “toll-lie”.

**********

PLOS authors have the option to publish the peer review history of their article (what does this mean?). If published, this will include your full peer review and any attached files.

If you choose “no”, your identity will remain anonymous but your review may still be made public.

**Do you want your identity to be public for this peer review?** For information about this choice, including consent withdrawal, please see our Privacy Policy.

Reviewer #1: No

Reviewer #2: No

Figure Files:

While revising your submission, please upload your figure files to the Preflight Analysis and Conversion Engine (PACE) digital diagnostic tool, https://pacev2.apexcovantage.com. PACE helps ensure that figures meet PLOS requirements. To use PACE, you must first register as a user. Then, login and navigate to the UPLOAD tab, where you will find detailed instructions on how to use the tool. If you encounter any issues or have any questions when using PACE, please email us at figures@plos.org.

Data Requirements:

Please note that, as a condition of publication, PLOS' data policy requires that you make available all data used to draw the conclusions outlined in your manuscript. Data must be deposited in an appropriate repository, included within the body of the manuscript, or uploaded as supporting information. This includes all numerical values that were used to generate graphs, histograms etc.. For an example see here on PLOS Biology: http://www.plosbiology.org/article/info%3Adoi%2F10.1371%2Fjournal.pbio.1001908#s5.

Reproducibility:

To enhance the reproducibility of your results, PLOS recommends that you deposit laboratory protocols in protocols.io, where a protocol can be assigned its own identifier (DOI) such that it can be cited independently in the future. For instructions see http://journals.plos.org/plospathogens/s/submission-guidelines#loc-materials-and-methods

# Author comment: Author response to Decision Letter 0

**DOI:** 10.1371/journal.ppat.1009278.r002

11 Jun 2021

Attachment Submitted filename: Response to Reviewer comments-final.docx Click here for additional data file.

# Editor report: Decision Letter 1

**DOI:** 10.1371/journal.ppat.1009278.r003

**Authors:** Guido Silvestri, Thomas J. Hope

15 Jun 2021

Dear Professor Gale Jr.,

We are pleased to inform you that your manuscript 'Interleukin-15 response signature predicts RhCMV/SIV vaccine efficacy' has been provisionally accepted for publication in PLOS Pathogens.

Before your manuscript can be formally accepted you will need to complete some formatting changes, which you will receive in a follow up email. A member of our team will be in touch with a set of requests.

Please note that your manuscript will not be scheduled for publication until you have made the required changes, so a swift response is appreciated.

IMPORTANT: The editorial review process is now complete. PLOS will only permit corrections to spelling, formatting or significant scientific errors from this point onwards. Requests for major changes, or any which affect the scientific understanding of your work, will cause delays to the publication date of your manuscript.

Should you, your institution's press office or the journal office choose to press release your paper, you will automatically be opted out of early publication. We ask that you notify us now if you or your institution is planning to press release the article. All press must be co-ordinated with PLOS.

Thank you again for supporting Open Access publishing; we are looking forward to publishing your work in PLOS Pathogens.

Best regards,

Guido Silvestri

Associate Editor

PLOS Pathogens

Thomas Hope

Section Editor

PLOS Pathogens

Kasturi Haldar

Editor-in-Chief

PLOS Pathogens

​orcid.org/0000-0001-5065-158X

Michael Malim

Editor-in-Chief

PLOS Pathogens

orcid.org/0000-0002-7699-2064

***********************************************************

Reviewer Comments (if any, and for reference):

# Editor report: Acceptance letter

**DOI:** 10.1371/journal.ppat.1009278.r004

**Authors:** Guido Silvestri, Thomas J. Hope

30 Jun 2021

Dear Professor Gale Jr.,

We are delighted to inform you that your manuscript, "Interleukin-15 response signature predicts RhCMV/SIV vaccine efficacy," has been formally accepted for publication in PLOS Pathogens.

We have now passed your article onto the PLOS Production Department who will complete the rest of the pre-publication process. All authors will receive a confirmation email upon publication.

The corresponding author will soon be receiving a typeset proof for review, to ensure errors have not been introduced during production. Please review the PDF proof of your manuscript carefully, as this is the last chance to correct any scientific or type-setting errors. Please note that major changes, or those which affect the scientific understanding of the work, will likely cause delays to the publication date of your manuscript. Note: Proofs for Front Matter articles (Pearls, Reviews, Opinions, etc...) are generated on a different schedule and may not be made available as quickly.

Soon after your final files are uploaded, the early version of your manuscript, if you opted to have an early version of your article, will be published online. The date of the early version will be your article's publication date. The final article will be published to the same URL, and all versions of the paper will be accessible to readers.

Thank you again for supporting open-access publishing; we are looking forward to publishing your work in PLOS Pathogens. 

Best regards,

Kasturi Haldar

Editor-in-Chief

PLOS Pathogens

​orcid.org/0000-0001-5065-158X

Michael Malim

Editor-in-Chief

PLOS Pathogens

orcid.org/0000-0002-7699-2064
